# Rituximab remodels the circulating immune landscape in pemphigus vulgaris revealed by high-dimensional immune profiling

**DOI:** 10.1016/j.jtauto.2026.100390

**Published:** 2026-08-03

**Authors:** Anne-Lise Strandmoe, Carlo G. Bonasia, Nanthicha Inrueangsri, Wayel H. Abdulahad, Inge M. Strating, Kevin P. Mennega, Theo Bijma, Gilles F.H. Diercks, Jeroen Bremer, Jon D. Laman, Barbara Horváth, Peter Heeringa

**Affiliations:** a Department of Pathology and Medical Biology, University of Groningen, University Medical Centre Groningen, Groningen, the Netherlands; b Department of Dermatology, University of Groningen, University Medical Centre Groningen, Centre for Blistering Diseases, Groningen, the Netherlands; c Department of Rheumatology and Clinical Immunology, University Medical Center Groningen, University of Groningen, Groningen, the Netherlands; d Department of Clinical Sciences, College of Medicine, University of Sharjah, M27 University City Road, Sharjah, United Arab Emirates

**Keywords:** Spectral flow cytometry, Pemphigus vulgaris, Rituximab, B cells, Autoimmunity

## Abstract

*Pemphigus vulgaris* (PV) is a severe autoimmune blistering disease characterized by pathogenic autoantibodies primarily targeting desmoglein-3. B-cell depletion by rituximab (anti-CD20) is an effective first-line treatment for PV; however, relapses are frequently observed. Evidence suggests that PV is associated with immune dysregulation beyond the B-cell compartment. So far, existing studies have largely focused on selected immune components or populations, or specific clinical states such as active disease, remission, or immediate post-rituximab, leaving it unclear how prior rituximab treatment shapes the overall circulating immune landscape during active PV, including relapse.

In this study, we performed high-dimensional immune profiling to characterize circulating immune-cell composition and cytokine/chemokine signatures using a 40-colour spectral flow cytometry panel and a 46-plex Luminex assay, respectively. We compared active PV patients with healthy controls and performed a subgroup analysis of rituximab-naive versus rituximab-experienced relapsed PV patients.

High-dimensional immune profiling showed that active PV is associated with a redistribution of circulating immune cells, with increased monocytes and decreased lymphocytes and plasmacytoid dendritic cells, together with reduced frequencies of double-negative (CD4^−^CD8^−^) T cells and T follicular helper cells compared with healthy controls. When comparing rituximab-naive with rituximab-experienced PV patients, in rituximab-experienced patients, the cellular composition changes were confined to the B-cell compartment, showing a shift toward a naive-dominant B-cell subset distribution. In parallel, PV was characterized by elevated serum concentrations of CD40L, CCL11/eotaxin, G-CSF, IL-1RA, IL-7, CCL20/MIP-3α, PD-L1/B7-H1/CD274, PDGF-AB/BB, and CCL5/RANTES, while no differences were identified between rituximab-naive patients and rituximab-experienced relapsed PV patients. Correlation analysis revealed that prednisone dosage may increase the frequency of mature B cells, and levels of CCL11/eotaxin, and IL-7, while decreasing the frequency of plasmacytoid dendritic cells and T follicular helper cells.

Together, these findings reveal a systemic immune signature of active PV involving changes in circulating monocyte, T-cell, and soluble immune compartments, whereas prior rituximab exposure in relapsed patients is associated with sustained remodelling confined to B-cell subset composition, without long-term restructuring of non–B-cell immune lineages.

## Graphical abstract

**Figure:**
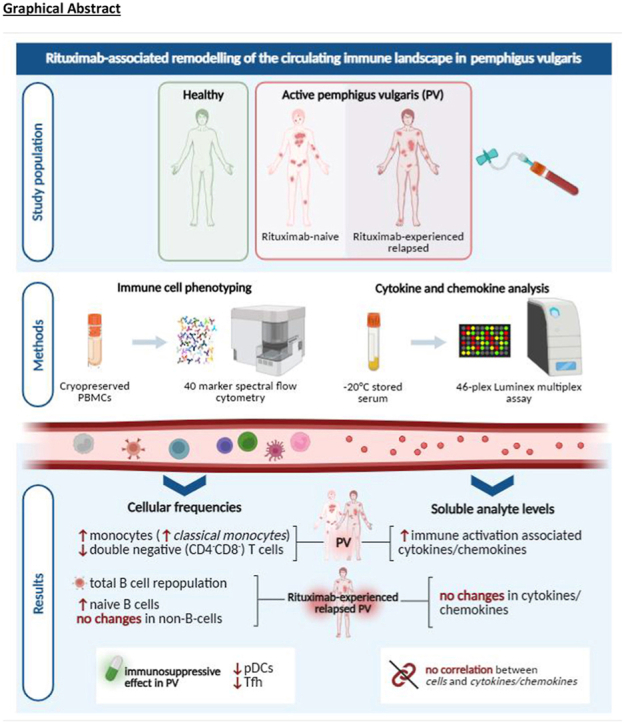

## 1 Introduction

*Pemphigus vulgaris* (PV) is a rare, chronic, and potentially life-threatening autoimmune blistering disease mediated by IgG autoantibodies targeting desmogleins (DSG), key cell adhesion proteins within desmosomes, resulting in intraepidermal acantholysis and blistering of the skin and/or mucous membranes [1,2]. Clinically, pemphigus presents in two subtypes: mucosal PV (mPV) and mucocutaneous PV (mcPV). In mPV, blistering is confined to mucosal surfaces and is primarily driven by anti-DSG3 autoantibodies, whereas mcPV involves both the skin and mucosa because of antibodies targeting both DSG1 and DSG3 [1].

PV is considered a prototypic antibody-mediated autoimmune disease [3,4]. The well-defined autoantigen targets and the ability of patient-derived IgG to reproduce key disease features in experimental models [5] render this condition valuable for exploring mechanisms of tolerance loss, immune network interactions, and the immunological effects of targeted B-cell therapies.

Therapy for PV was revolutionized by rituximab, a monoclonal anti-CD20 B-cell–depleting agent, which dramatically improves disease outcome. DSG3-specific B cells are central to PV pathogenesis, and their depletion by rituximab underlies its clinical effectiveness [[6], [7], [8], [9], [10]]. However, relapse following rituximab treatment is a major clinical challenge, affecting approximately half of treated patients [11], and remains difficult to predict using current immune monitoring approaches that rely primarily on B-cell counts and autoantibody titers.

Although PV is primarily driven by pathogenic autoantibodies, both disease pathogenesis and therapeutic response involve broader immune dysregulation beyond autoreactive B cells. Multiple studies implicate T cells in both PV pathogenesis and immune modulation following rituximab treatment. DSG-specific CD4^+^ T-cell subsets contribute to B-cell-mediated autoantibody production and disease activity [12], and increased frequencies of T helper (Th) [[13], [14], [15], [16], [17]] and reduced frequency of regulatory T-cell (Treg) populations [16] have been reported in PV. Rituximab treatment is associated with changes in circulating T-cell subsets, including a decrease in T follicular helper (Tfh) cells and an emergence of DSG3-specific follicular regulatory (Tfr) cells [18,19]. In parallel, components of the innate immune system have also been implicated in PV. Distorted frequencies and functional changes in innate lymphoid cells (ILCs), natural killer (NK) cells, dendritic cells (DCs), and NK-T cells have been reported and linked to PV disease or rituximab treatment response compared to a healthy cohort [[20], [21], [22], [23], [24]]. However, how rituximab affects the innate immune compartment in PV remains largely unexplored.

So far, published studies have largely focused on individual immune populations or isolated clinical states such as active disease, remission, or immediate post-rituximab. Consequently, the extent to which rituximab treatment shapes the broader circulating immune landscape in patients with active PV, regardless of relapse status or prior treatment exposure, has yet to be determined. To address this, we performed high-dimensional immune profiling comparing active disease PV patients with healthy controls (HCs) and, within the PV cohort, comparing rituximab-naive patients with rituximab-experienced relapsed patients. By integrating high-dimensional cellular phenotyping with circulating cytokine and chemokine profiling, this study investigated the immunological footprint of prior rituximab treatment in relapsed rituximab-treated PV patients. This approach may provide a basis for extending immune monitoring and treatment beyond B cells.

## 2 Materials and methods

### 2.1 Study population

A single-centre prospective study was performed, including patients with PV at the University Medical Center Groningen (UMCG) Center of Expertise for Blistering Diseases, in The Netherlands. Written informed consent was obtained from all participants, and the study was approved by the Institutional Review Board of the University Medical Center Groningen (METc no. 2012/151 and 2020/007). PV diagnosis was confirmed based on clinical presentation and histopathology findings, direct immunofluorescence showing intercellular deposits of IgG and/or C3, and positive anti-DSG1 and/or anti-DSG3 IgG by ELISA [25]. All PV patients had active disease; peripheral blood was sampled at baseline, defined as the day of rituximab initiation and prior to the first rituximab infusion. At the time of sampling, patients were managed according to routine clinical practice and international PV treatment guidelines. Prednisone had generally been initiated at diagnosis at 0.5–1.0 mg/kg/day, depending on disease severity, followed by tapering over approximately 6–8 weeks. Consequently, prednisone dose at sampling varied between patients depending on disease severity and the interval between prednisone initiation and rituximab treatment. Patients tested positive for anti-DSG3 antibodies and had no clinically relevant comorbid conditions expected to substantially affect circulating immune profiles, including other autoimmune, active infectious, malignant, or systemic inflammatory diseases. PV patients were divided into rituximab-naive (nPV) and rituximab-non-naive (nnPV) groups, the latter defined as patients who had previously received one or more rituximab cycles and subsequently relapsed; rituximab-experienced relapsed patients were sampled approximately one year or more after their last rituximab administration, with ranges of 14–59 months in the spectral flow cytometry cohort and 11–62 months in the Luminex cohort. HCs were age- and sex-matched to the PV cohort. Controls had no known history of pemphigus or other autoimmune disease, active inflammatory or infectious disease, malignancy, or systemic immunosuppressive treatment at the time of sampling. Skin inspection was performed as part of the clinical assessment. Clinical characteristics collected include sex, age at sampling, PV subtype (mPV or mcPV), anti-DSG1 and anti-DSG3 titre, routine CD19^+^ B cells concentrations, total disease duration between the moment of onset of first lesions to the point of sampling, pemphigus area disease index (PDAI) score, time between last rituximab administration and time of sampling, immunosuppressive medication, and comorbidities at time of sampling. A separate HC cohort was used for spectral flow cytometry analysis and Luminex assays. The same PV patients and time points were used across both platforms, with five additional patients included in the Luminex dataset.

### 2.2 PBMC and serum isolation

**Fig. 1:**
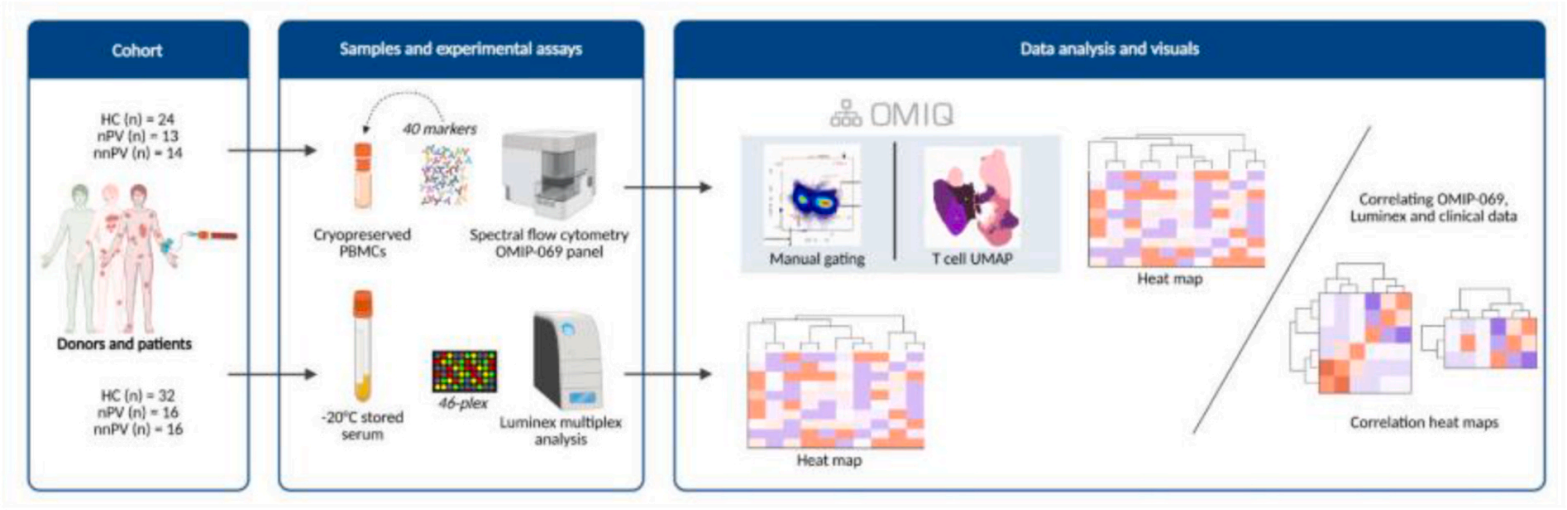
**Overview of the experimental workflow,** depicting patient groups, sample sizes, experimental assays, and subsequent data processing steps such as manual gating, UMAP visualization, heatmap generation, and correlation analyses**.** Abbreviations: HC, healthy control; nPV, rituximab naive pemphigus vulgaris; nnPV, rituximab non-naive pemphigus vulgaris; PBMC, peripheral blood mononuclear cells; OMIP-069, optimized multicolour immunofluorescence panel; UMAP, uniform manifold approximation and projection.

Peripheral blood was collected in lithium-heparin-coated tubes. Peripheral blood mononuclear cells (PBMCs) were directly isolated using Lymphoprep (Stemcell, Vancouver, Canada) density-gradient centrifugation. The isolated PBMCs were cryopreserved by freezing in a controlled manner to −80°C in Roswell Park Memorial Institute 1640 medium (RPMI 1640, Lonza, Basel, Switzerland) supplemented with 10% fetal bovine serum (FBS, Lonza), 10% dimethyl sulfoxide, and 50 μg/mL gentamycin (Lonza) before being stored in liquid nitrogen. Serum samples were isolated from silica-coagulated blood and stored at −20°C. PBMCs and serum samples were used for spectral flow cytometry and the Luminex multiplex assay, respectively (Fig. 1).

### 2.3 Spectral flow cytometry

Antibody staining of PBMC samples for spectral flow cytometry was performed according to the OMIP-069 protocol with minor modifications, as described by Bonasia et al. [26]. Cryopreserved PBMCs were thawed in a 37°C water bath, washed twice in complete RPMI (RPMI 1640 medium supplemented with 10% FBS and 50 μg/mL gentamycin), and resuspended in complete RPMI at a concentration of 3.5 × 10^6^ cells/mL. For each sample, 2 × 10^6^ PBMCs were washed with PBS and stained with a 1:40 dilution of viability dye (LIVE/DEAD Fixable Blue Stain; Thermo Fisher Scientific, Waltham, MA, USA) for 15 min. After washing with PBS, the cells were incubated with Brilliant Stain Buffer Plus (BD Biosciences, San Jose, USA), True-Stain Monocyte Blocker (BioLegend, San Diego, CA, USA), and anti-TCRγδ (PerCP-eFluor710, clone B1.1; Thermo Fisher Scientific) for 10 min; followed by sequential staining with anti-CCR5 (BUV563, clone 2D7/CCR5; BD Biosciences) and anti-CXCR5 (BV750, clone RF8B2; BD Biosciences) for 10 min, and anti-IgG (BV605, clone G18-145; BD Biosciences) for 10 min. The remaining antibodies were added as a cocktail and incubated for 30 min (Supplementary Table 1). Samples were washed with PBS containing 1% BSA and fixed for 20 min in 1:10 diluted FACS Lysing Solution (BD Biosciences). Finally, the samples were washed and resuspended in PBS. All staining steps were performed in the dark at room temperature. For measurement, 1 × 10^6^ PBMCs per sample were acquired using a 5-laser CYTEK Aurora full-spectrum flow cytometer (CYTEK Biosciences, Fremont, CA, USA). Samples were randomly distributed across batches with a balanced inclusion of HC and PV samples to minimize batch effects. Unstained and single-stained control samples were used for antibody validation.

### 2.4 Flow cytometry data analysis by manual gating

Flow cytometry data were analysed using OMIQ software (www.omiq.ai, Dotmatics, Boston, MA, USA). Manual gating strategies for identifying circulating immune cell subsets were adapted from OMIP-069 [27] and Bonasia et al. [26] (Supplementary Fig. 1). To assess data quality and potential batch effects, samples were grouped by batch, and marker histograms were plotted to check for deviations. The absolute counts (cells ×10^6^/L) of basophils, lymphocytes, and monocytes were derived from routine clinical data from the UMCG diagnostics laboratory. *Main immune subset* counts were calculated by multiplying the absolute count of the parent population by the *main immune subset* frequency from flow cytometry. Frequencies were expressed both as a percentage of live/CD45^+^ PBMCs and relative to their parent population.

### 2.5 High-dimensional data reduction

Unsupervised dimensionality reduction and visualization of T cell spectral flow cytometry data were performed using OMIQ software by applying the uniform manifold approximation and projection (UMAP) algorithm. From the live-cell manual gate, 7,066,050 cells were subsampled across 51 samples (138,550 cells per sample) and the quality control was performed using the FlowCut algorithm. From the CD3^+^TCRγδ ^±^ gate, 1,656,837 T cells were selected, aiming for 34,482 cells (minimum 10,000 cells) to achieve approximately equal cell counts across samples for most samples. Subsequently, UMAP analysis was performed to visualize different T-cell subpopulations. UMAP was optimized using the following settings: 51 files used (HC = 24; PV = 27), 10 fluorescent markers (CD45RA-BUV395, CD8-BUV805, CCR7-BV421, CD28-BV650, PD-1-BV785, TCRγδ-PerCP-eFluor 710, CD4-cFluor YG584, CD25-PE-Alexa Fluor 700, CD127-APC-R700, and CD27-APC-H7), neighbors = 5, minimum distance = 0.4, components = 2, metric = Euclidean, learning rate = 1, epochs = 600, random seed = 2612, embedding initialization = spectral. FlowSOM was then run to cluster the data with the following settings: 10 fluorescent features (CD45RA-BUV395, CD8-BUV805, CCR7-BV421, CD28-BV650, PD-1-BV785, TCRγδ-PerCP-eFluor 710, CD4-cFluor YG584, CD25-PE-Alexa Fluor 700, CD127-APC-R700, CD27-APC-H7), 20 clusters with xdim = 15 and ydim = 15, rlen = 10, Distance Metric = Euclidean, consensus metaclustering with k = 20, Random Seed = 2612. Similar clusters from FlowSOM were merged based on T cell main marker expression (CD4, CD8, TCRγδ, CD45RA, CD27, CD28, CCR7), resulting in 10 final clusters. A heatmap was generated using hierarchical clustering based on Euclidean distance to visualize marker similarities across populations. FlowSOM clusters were manually annotated based on marker expression described in existing literature [[28], [29], [30]] and mapped onto the UMAP to compare the HC, nPV, and nnPV groups. The counts of each cluster were extracted from the UMAP.

### 2.6 Cytokine/chemokine profiling

Circulating levels of 46 cytokines and chemokines (Supplementary Table 2) were measured using the Human XL Cytokine Luminex Performance Panel Premixed Kit (R&D Systems, Minneapolis, MN, USA) following the manufacturer's instructions. Plates were read on a Luminex 200 detection system (Luminex, Austin, TX, USA) and data were processed using xPONENT software version 4.2 (DiaSorin, Saluggia, Italy). Data points with bead counts below 50 were excluded. Cytokines or chemokines with > 20% missing data owing to low bead counts were also removed. For the included cytokines and chemokines, values below the standard curve were set to 0.5 times the lowest standard, and values above the standard curve were set to 1.5 times the highest standard curve value.

### 2.7 Statistical analysis and data visualization

Manual gating, high-dimensional data reduction, and Luminex data were analysed and graphed using GraphPad Prism version 10.4.0 (GraphPad Software, La Jolla, CA, USA) and RStudio version 4.4.2. Heatmaps, correlation analyses, and plotting were generated using RStudio version 4.4.2. Comparative analyses were conducted at two levels: initially, between HCs and PV patients, and subsequently within the PV cohort, to assess differences between the nPV and nnPV subgroups. Analyses were performed on the following datasets: manually gated *major immune cell populations* within the CD45^+^ compartment (% of CD45^+^ cells), *main immune cell subsets* within *major immune cell populations* (% of their respective major parent population), absolute counts of *major immune cell populations* in PV samples (nPV vs. nnPV only), and T-cell clusters identified by high-dimensional reduction (% of total T cells). The 12 *major immune cell populations* are broad cellular compartments, and the 62 *main immune cell subsets* are sub-compartments within major immune cell population compartments (Supplementary Table 3). Group differences were assessed using the Mann-Whitney *U* test. Multiple testing was controlled for using the Benjamini-Hochberg (BH) procedure to control the false discovery rate. Spearman's rank correlation coefficient was used to explore correlations between immune cell populations, cytokines/chemokines, and clinical parameters. Heatmaps were generated using Ward's agglomerative hierarchical clustering. BH-adjusted p-values < 0.05 were considered statistically significant.

## 3 Results

### 3.1 Patient characteristics

In the spectral flow cytometry analysis, 27 PV patients (nPV, n = 13; nnPV, n = 14) and 24 HCs were included. In the Luminex multiplex assay, 32 PV patients (nPV, n = 16; nnPV, n = 16) and 32 HCs were included. The clinical characteristics are presented in Table 1. Most patients received immunosuppressive therapy with prednisone as part of standard care.

**Table 1:** Patient and healthy control characteristics. Abbreviations: HC, healthy control; nPV, rituximab-naive pemphigus vulgaris; nnPV, rituximab-non-naive pemphigus vulgaris; mPV, mucosal dominant pemphigus vulgaris; mcPV; mucocutaneous pemphigus vulgaris; anti-DSG3, desmoglein-3 targeting antibodies; anti-DSG1, desmoglein-1 targeting antibodies; PDAI, pemphigus disease area index; RTX; rituximab.

	Spectral flow cytometry			Luminex multiplex assay		
	HC	nPV	nnPV	HC	nPV	nnPV
**Total, n**	24	13	14	32	16	16
**Sex (% male)**	58	62	36	53	63	38
**Age, mean (range)**	60 (34 - 86)	55 (32 - 71)	61 (37 - 87)	58 (30 - 75)	56 (32 - 71)	60 (37 - 87)
**mPV patients, n**	-	7 (54)	9 (64)	-	7 (44)	10 (63)
**mcPV patients, n**	-	6 (46)	5 (36)	-	9 (56)	6 (38)
**anti-DSG1 titre (U/mL), median (range)**	-	3 (0 - 270)	8 (0 - 750)	-	6 (0 - 270)	8 (0 - 750)
**anti-DSG3 titre (U/mL), median (range)**	-	455 (39 - 5600)	69 (4 - 750)	-	455 (39 - 5600)	89 (16 - 750)
**CD19^+^ B cells (x 10^9^ U/L), median (range)**	-	0.20 (0.04 - 0.36)	0.27 (0.04 - 0.65)	-	0.22 (0.04 - 0.36)	0.26 (0.02 - 0.65)
**Disease duration (months), median (range)**	-	11 (3 - 241)	71 (39 - 230)	-	9 (3 - 241)	71 (30 - 230)
**PDAI, median (range)**	-	8 (2 - 22)	7 (1 - 16)	-	7 (0 - 24)	2 (0 - 115)
**Time since last RTX administration (months), median (range)**	-	-	30 (14 - 59)	-	-	30 (11 - 62)
**Concomitant therapy**^a^	-	-	-	-	-	-
**Prednisone, n (%)**	-	8 (62)	3 (21)	-	11 (69)	5 (31)
**Dosage (mg/day), median (range)**	-	33 (25 - 100)	50 (20 - 60)	-	40 (25 - 100)	50 (20 - 60)
**Azathioprine, n (%)**	-	1 (8)	1 (7)	-	1 (6)	1 (6)
**Dosage (mg/day), median (range)**	-	150 (150 - 150)	150 (150 - 150)	-	150 (150 - 150)	-
**Other immunosuppressants, n (%)**	-	1 (8)	0 (0)	-	1 (6)	0 (0)
**Comorbidities, n (%)**	-	3 (23)	7 (50)	-	4 (25)	7 (44)

a Immunosuppressive medication at blood sampling time point.

### 3.2 *Pemphigus vulgaris* is associated with altered frequencies of circulating monocyte and T-cell populations

To characterize the distribution of immune cells within circulating PBMCs from PV patients and HCs, frequencies of *major immune cell populations* (Supplementary Table 4, Supplementary Fig. 2) and the *main immune cell subsets* (Supplementary Table 5, Supplementary Fig. 3) were quantified by spectral flow cytometry.

**Fig. 2:**
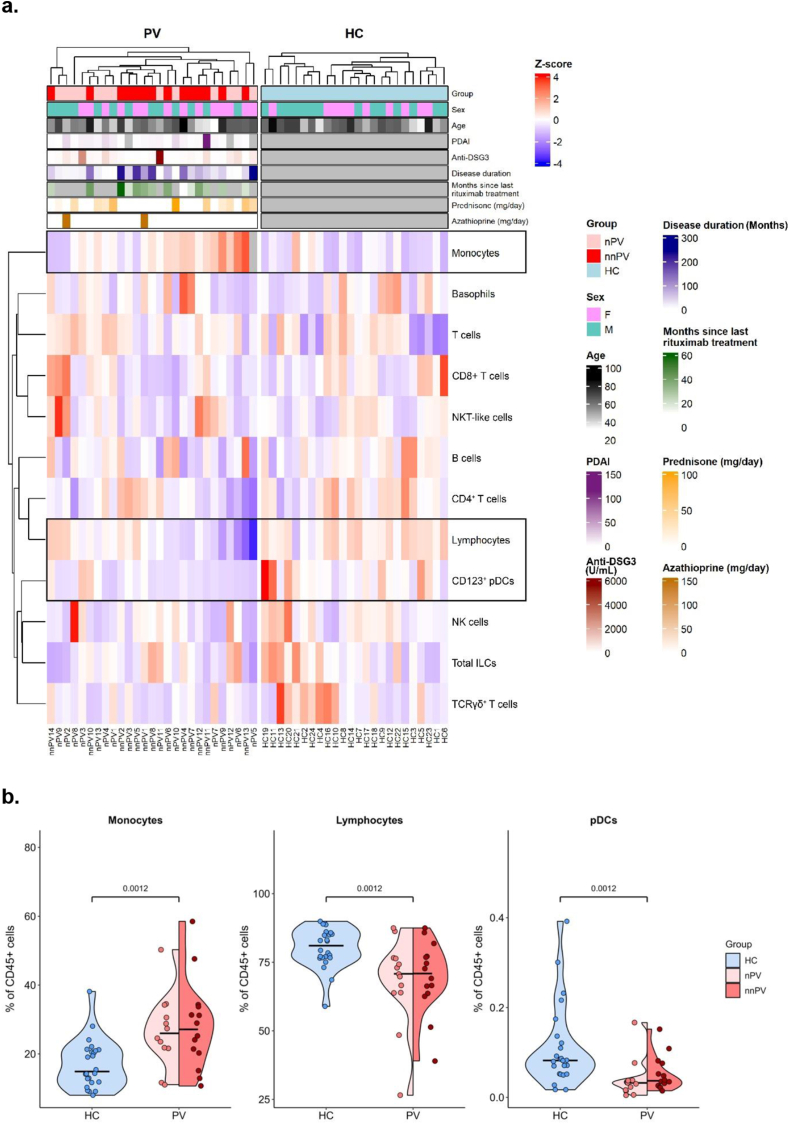
**Frequencies of manually gated major immune cell populations. a.** Heatmap of the frequencies of manually gated major populations within the CD45^+^ cell population and basophil frequency within the live cell population in rituximab-naive (nPV) and rituximab-non-naive (nnPV) pemphigus vulgaris patients, and healthy controls (HCs). Ward's hierarchical agglomerative clustering was applied to generate the heatmap. Cell frequencies were transformed into z-scores, with the blue-red colour scale representing low to high frequency, respectively. Clinical parameters are depicted above the heatmap and include sex, age, PDAI score, anti-DSG3 antibody titre, total disease duration in months, time since last rituximab treatment, prednisone dosage, and azathioprine dosage. Immune subsets with significantly different frequencies are highlighted by a black rectangle. **b.** Violin plots showing the frequencies of the manually gated monocytes, lymphocytes, and plasmacytoid dendritic cells (pDCs) within the CD45^+^ compartment. Comparisons were performed between healthy controls (HCs), pemphigus vulgaris (PV) patients, and the subgroups of rituximab-naive (nPV) and rituximab-non-naive (nnPV) patients. Mann-Whitney U tests were used, and p-values were adjusted for multiple comparisons using the Benjamini-Hochberg false discovery rate correction. Significant differences (adjusted P < 0.05) are indicated on the plots. Violin plots showing the frequencies of all measured major immune cell populations are provided in Supplementary Fig. 2. Abbreviations: PV, pemphigus vulgaris; HC, healthy control; NK, natural killer; pDC, plasmacytoid dendritic cell; ILC, innate lymphoid cell; TCRgd, T-cell receptor γδ; PDAI, pemphigus disease area index; DSG, desmoglein.

**Fig. 3:**
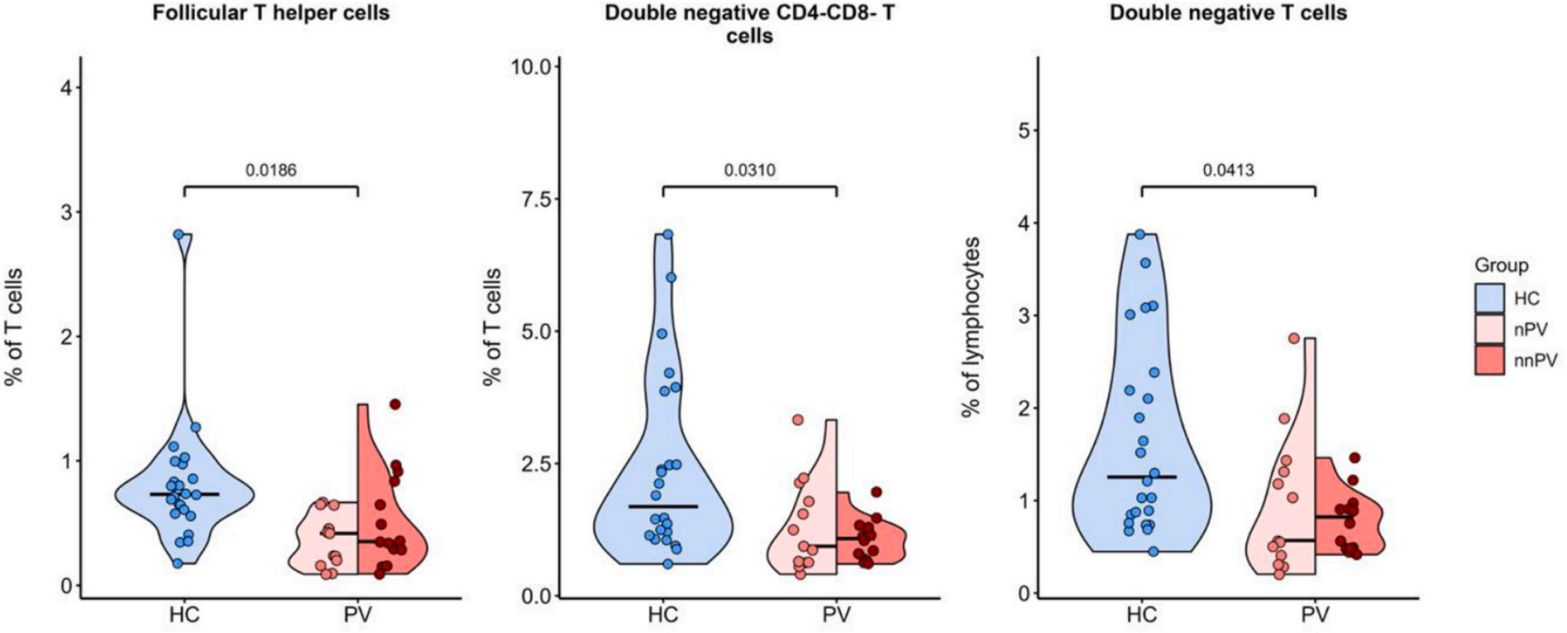
– **Frequencies of manually gated T cell subsets within the total T cell population and lymphocytes.** Violin plots showing the frequencies of manually gated follicular helper T cells and double-negative (CD4^−^CD8^−^) T cells within the T-cell population, as well as double-negative (CD4^−^CD8^−^) T cells within the lymphocyte population. Comparisons were performed between healthy controls (HCs), pemphigus vulgaris (PV) patients, and the subgroups of rituximab-naive (nPV) and rituximab-non-naive (nnPV) patients. Mann-Whitney U tests were used, and p-values were adjusted for multiple comparisons using the Benjamini-Hochberg false discovery rate correction. Significant differences (adjusted P < 0.05) are indicated on the plots. Abbreviations: HC, healthy control; PV, pemphigus vulgaris; nPV, rituximab-naive PV; nnPV, rituximab-non-naive PV.

A comparative analysis between patients with PV and HCs revealed that patients with PV demonstrated an increased frequency of monocytes within the CD45^+^ compartment compared with HCs (Fig. 2). Within the monocyte population, the *main* monocyte *subsets* showed a trend toward increased classical monocytes and reduced intermediate and non-classical monocytes in PV compared with HCs; these differences did not remain statistically significant after BH correction (Supplementary Table 5). Additionally, within the CD45^+^ PBMC compartment, patients with PV demonstrated decreased frequencies of lymphocytes and plasmacytoid dendritic cells (pDCs) compared with HCs (Fig. 2). Examination of the *main* T-cell *subsets* showed a reduction in double-negative T cells and Tfh cells in PV patients compared to HCs. Consistently, CD4^−^CD8^−^ T cells were observed at a lower frequency when analysed as a proportion of the total lymphocyte population (Fig. 3). However, unsupervised UMAP analysis also did not indicate broad restructuring of the T-cell landscape between PV patients and HCs (Supplementary Table 8, Supplementary Fig. 9). To assess whether the observed PV-associated changes in major immune cell populations were influenced by systemic immunosuppressive treatment, we performed a subgroup analysis restricted to PV patients not receiving systemic immunosuppressive medication at the time of sampling. This analysis showed a directionally similar pattern for major immune-cell populations, with increased monocyte and decreased lymphocyte frequencies compared to HCs, although the pDC difference was less pronounced (Supplementary Fig. 4).

Together, these findings indicate that PV is associated with a redistribution of *major immune cell populations* and *main immune cell subsets*, most notably characterized by changes within the monocyte and T-cell compartments.

### 3.3 Prior rituximab treatment is associated with sustained remodelling of the circulating B-cell compartment in pemphigus vulgaris

To assess whether prior rituximab exposure influenced the circulating immune cell landscape in PV, we performed a subgroup comparison of manually gated *major immune cell populations* (Supplementary Table 4, Supplementary Fig. 2) and *main immune cell subset* frequencies (Supplementary Table 5, Supplementary Fig. 3) between nPV and nnPV patients within the PV cohort.

**Fig. 4:**
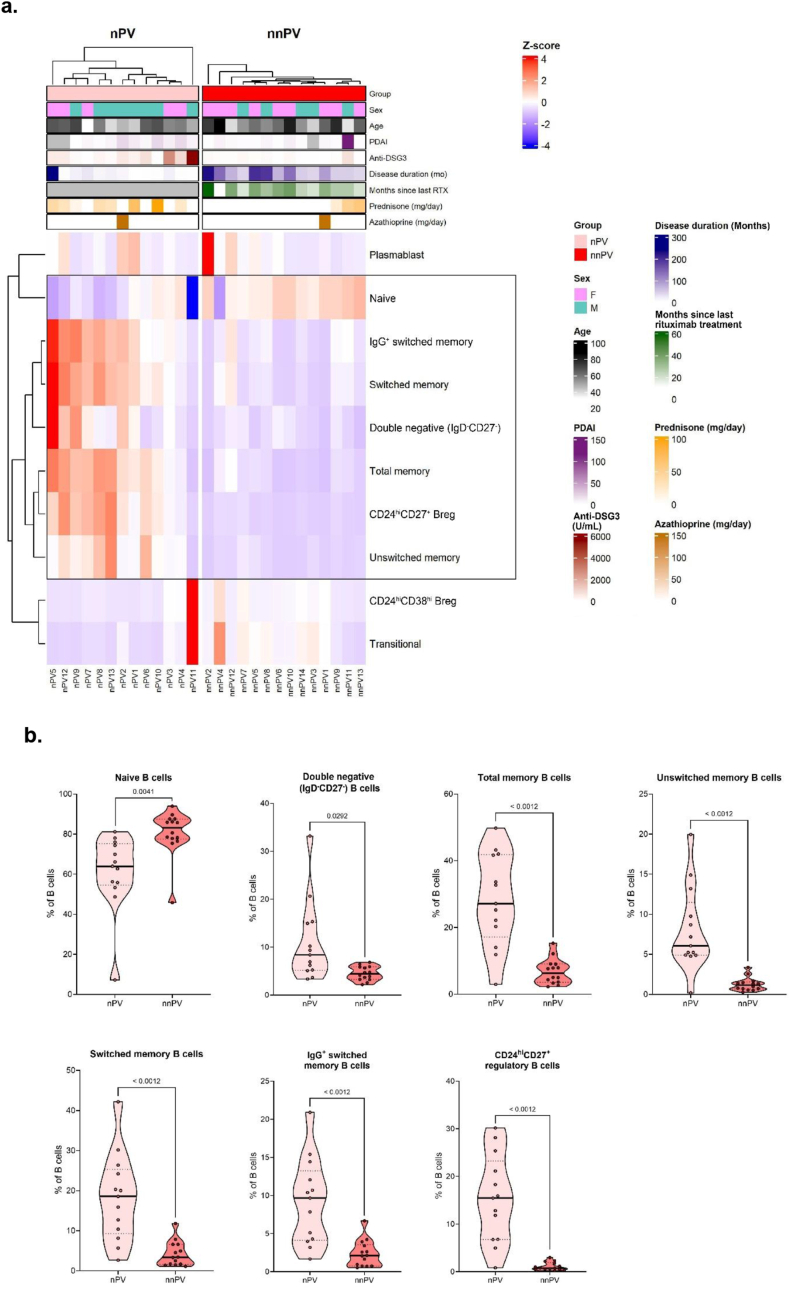
**Frequencies of manually gated B cell subsets within the total B cell population. a.** Heatmap showing the manually gated frequencies of B cell subsets within the total B cell population in rituximab-naive (nPV) and rituximab-non-naive (nnPV) pemphigus vulgaris patients. Ward's hierarchical agglomerative clustering was applied to generate the heatmap. Cell frequencies were transformed into z-scores, with the blue-red colour scale representing low to high frequency, respectively. Clinical parameters are depicted above the heatmap and include sex, age, PDAI score, anti-DSG3 antibody titre, total disease duration in months, months since last rituximab treatment, prednisone dosage, and azathioprine dosage. Immune subsets with significantly different frequencies are highlighted by a black rectangle. **b.** Violin plots showing the frequencies of naive B cells, double negative (IgD^−^CD27^−^) naive B cells, total memory B cells, unswitched memory B cells, switched memory B cells, IgG^+^ switched memory B cells, and CD24^hi^CD27^+^ regulatory B cells within the B-cell population. Mann-Whitney U tests were used, and p-values were adjusted for multiple comparisons using the Benjamini-Hochberg false discovery rate correction. Significant differences (adjusted P < 0.05) are indicated on the plots. Abbreviations: PV, pemphigus vulgaris; PDAI, pemphigus disease area index; DSG, desmoglein; RTX, rituximab; nPV, rituximab-naive PV; nnPV, rituximab-non-naive PV; Breg, regulatory B cell.

As expected, pronounced differences were identified within the *main* B-cell *subsets*. Compared with nPV patients, nnPV patients displayed a significantly higher proportion of naive B cells and lower frequencies of double-negative (IgD^−^CD27^−^) B cells, total memory B cells, unswitched and switched memory B cells, IgG^+^ switched memory B cells, and CD24^hi^CD27^+^ regulatory B cells within the total B-cell population (Fig. 4). Additionally, within the total B-cell population, trends toward reduced frequencies of transitional B cells and CD24^hi^CD38^hi^ regulatory B cells were observed in nPV compared with nnPV patients; these differences did not remain statistically significant after BH correction (Supplementary Table 5).

No significant differences between the nPV and nnPV patients were observed in the absolute counts of *major immune cell populations* (Supplementary Table 4, Supplementary Fig. 5) nor across non–B-cell lineages within the other manually gated *main immune cell subsets* (Supplementary Table 5, Supplementary Fig. 3). Additionally, unsupervised UMAP analysis did not indicate broad restructuring of the T-cell landscape between nPV and nnPV patients (Supplementary Table 8, Supplementary Fig. 9).

Collectively, these findings indicate that prior rituximab therapy is associated with a sustained shift towards a naive B cell subset composition in PV, without significant long-term effects on other *major* immune cell *populations* or *main subsets*.

### 3.4 *Pemphigus vulgaris* is associated with increased circulating cytokine and chemokine levels

**Fig. 5:**
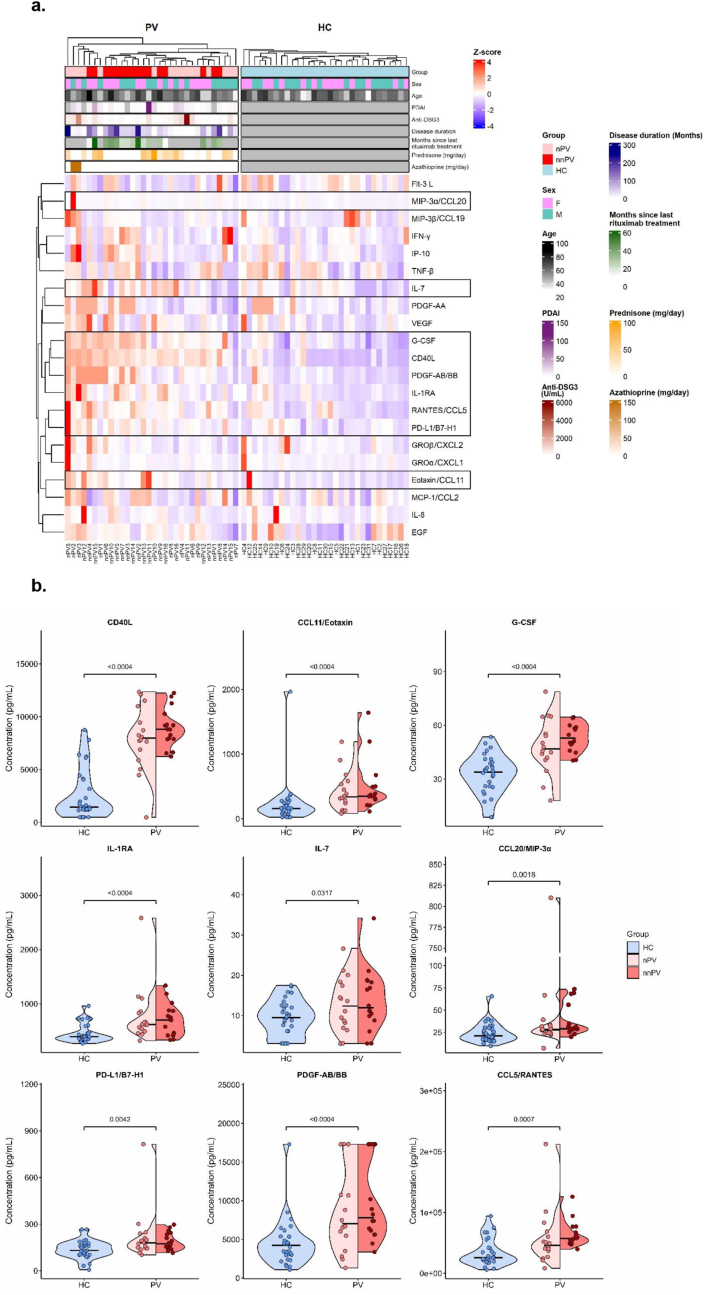
**Serum analyte concentrations. a.** Heatmap of serum analyte concentrations measured by Luminex. The heatmap was generated using Ward's hierarchical agglomerative clustering. Analyte concentrations were transformed into z-scores, with the blue-red colour scale representing low to high frequency, respectively. Clinical parameters are depicted above the heatmap and include sex, age, PDAI score, anti-DSG3 antibody titre, total disease duration in months, months since last rituximab treatment, prednisone dosage, and azathioprine dosage. Immune subsets with significantly different frequencies are highlighted by a black rectangle. **b.** Violin plots of serum concentrations of the nine analytes that were significantly different between PV patients and HCs. Concentrations were measured using the Human XL Cytokine Luminex Performance Assay 46-plex Fixed Panel. Mann-Whitney U tests were used, and p-values were adjusted for multiple comparisons using the Benjamini-Hochberg false discovery rate correction. Significant differences (adjusted P < 0.05) are indicated on the plots. Violin plots showing the serum concentrations of all measured analytes are provided in Supplementary Fig. 6. Abbreviations: PV, pemphigus vulgaris; HC, healthy control; PDAI, pemphigus disease area index; DSG, desmoglein; nPV, rituximab-naive PV; nnPV, rituximab-non-naive PV.

To further characterize systemic immune alterations in PV, serum samples from PV patients and HCs were analysed using the Human XL Cytokine Luminex Performance Assay 46-plex Fixed Panel. After quality control, 25 cytokines and chemokines with consistent detection across all samples were included in the quantitative analysis. Compared with HCs, PV patients exhibited significantly elevated serum concentrations of CD40L, CCL11/eotaxin, G-CSF, IL-1RA, IL-7, CCL20/MIP-3α, PD-L1/B7-H1/CD274, PDGF-AB/BB, and CCL5/RANTES (Fig. 5, Supplementary Table 7). Subgroup analysis of nPV and nnPV patients revealed no significant differences in the levels of these cytokines and chemokines (Supplementary Table 7, Supplementary Fig. 6).

Together, these findings indicate that PV is associated with increased circulating cytokines and chemokines linked to adaptive immune activation, including T-cell- and B-cell-related pathways (CD40L, PD-L1/B7-H1/CD274, IL-7, CCL5/RANTES, and CCL11/eotaxin), as well as innate immune recruitment (CCL11/eotaxin, G-CSF, CCL20/MIP-3α, and CCL5/RANTES), alongside elevated tissue remodelling associated growth factor PDGF-AB/BB.

### 3.5 Clinical parameters and immune alterations associate with coordinated B-cell subsets and specific cytokine/chemokine patterns in pemphigus vulgaris

**Fig. 6:**
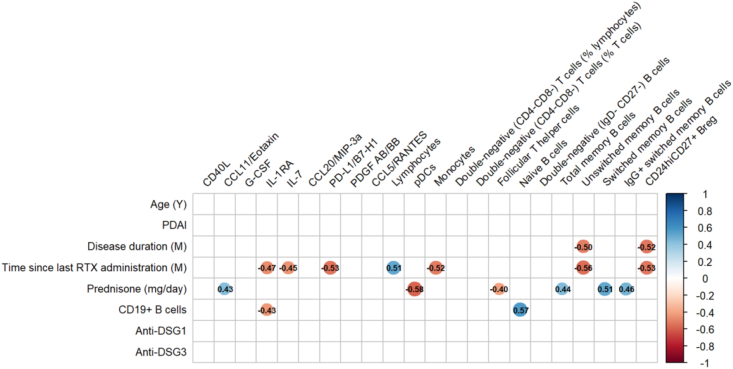
**Correlation between clinical parameters and immunological findings.** Heatmap demonstrating the correlations of the significantly different immune cell frequencies and serum concentrations of analytes in pemphigus vulgaris (PV) patients with clinical parameters. Circle size is proportional to the magnitude of the Spearman correlation coefficient, and correlations between −0.4 and 0.4 are not displayed. Correlations involving time since last rituximab administration were analysed only within the nnPV group. p-values were adjusted for multiple comparisons using the Benjamini-Hochberg false discovery rate correction. Full correlation statistics are provided in Supplementary File 1. Abbreviations: Y, years; RTX, rituximab; DSG, desmoglein; Breg, regulatory B cell; PV, pemphigus vulgaris; nPV, rituximab-naive PV; nnPV, rituximab-non-naive PV; PDAI, pemphigus disease area index; pDC, plasmacytoid dendritic cell; M, months.

To examine associations between significantly altered immune cell populations, circulating cytokines and chemokines, and clinical parameters in PV, correlation analyses were performed (Fig. 6, Supplementary Fig. 7, and Supplementary File 1). Several moderate correlations were observed, although these did not reach statistical significance after adjustment for multiple testing. Disease duration showed moderate negative correlations with the frequencies of unswitched memory B cells (r = −0.50) and CD24^hi^CD27^+^ regulatory B cells (r = −0.52), predominantly driven by values from nnPV patients (Supplementary Fig. 7). Similarly, in nnPV patients, time since last rituximab administration correlated with the frequencies of unswitched memory B cells (r = −0.56) and CD24^hi^CD27^+^ regulatory B cells (r = 0.53), consistent with B-cell depletion–driven reconstitution. Time since last rituximab administration also showed negative correlations with monocyte frequency (r = −0.52), IL-1RA (r = −0.47), IL-7 (r = −0.45), and PD-L1/B7-H1/CD274 (r = −0.53), while a positive correlation was observed with lymphocyte frequency (r = 0.51). Prednisone dosage showed multiple associations with immune cell subsets and soluble cytokines/chemokines. Higher prednisone doses at time of sampling correlated positively with serum CCL11/eotaxin (r = 0.48) and IL-7 (r = 0.41) levels, as well as with the frequencies of total memory B cells (r = 0.47), switched memory B cells (r = 0.56), IgG^+^ switched memory B cells (r = 0.51), and CD24^hi^CD27^+^ regulatory B cells (r = 0.42). In contrast, prednisone dosage correlated negatively with pDC (r = −0.59) and Tfh (r = −0.41) frequencies. Further associations were observed with circulating CD19^+^ B cell concentration. IL-1 R A levels correlated negatively (r = −0.43), whereas naive B-cell frequency correlated positively with CD19^+^ B-cells (r = 0.57). Additionally, naive B-cell frequency correlated negatively with anti-DSG3 antibody titers (r = −0.45), while CD24^hi^CD27^+^ regulatory B-cell frequency correlated positively with anti-DSG3 titers (r = 0.41).

Collectively, these findings indicate that clinical variables, notably corticosteroid exposure and the time since last rituximab administration, are associated with distinct immunological profiles in PV.

**Fig. 7:**
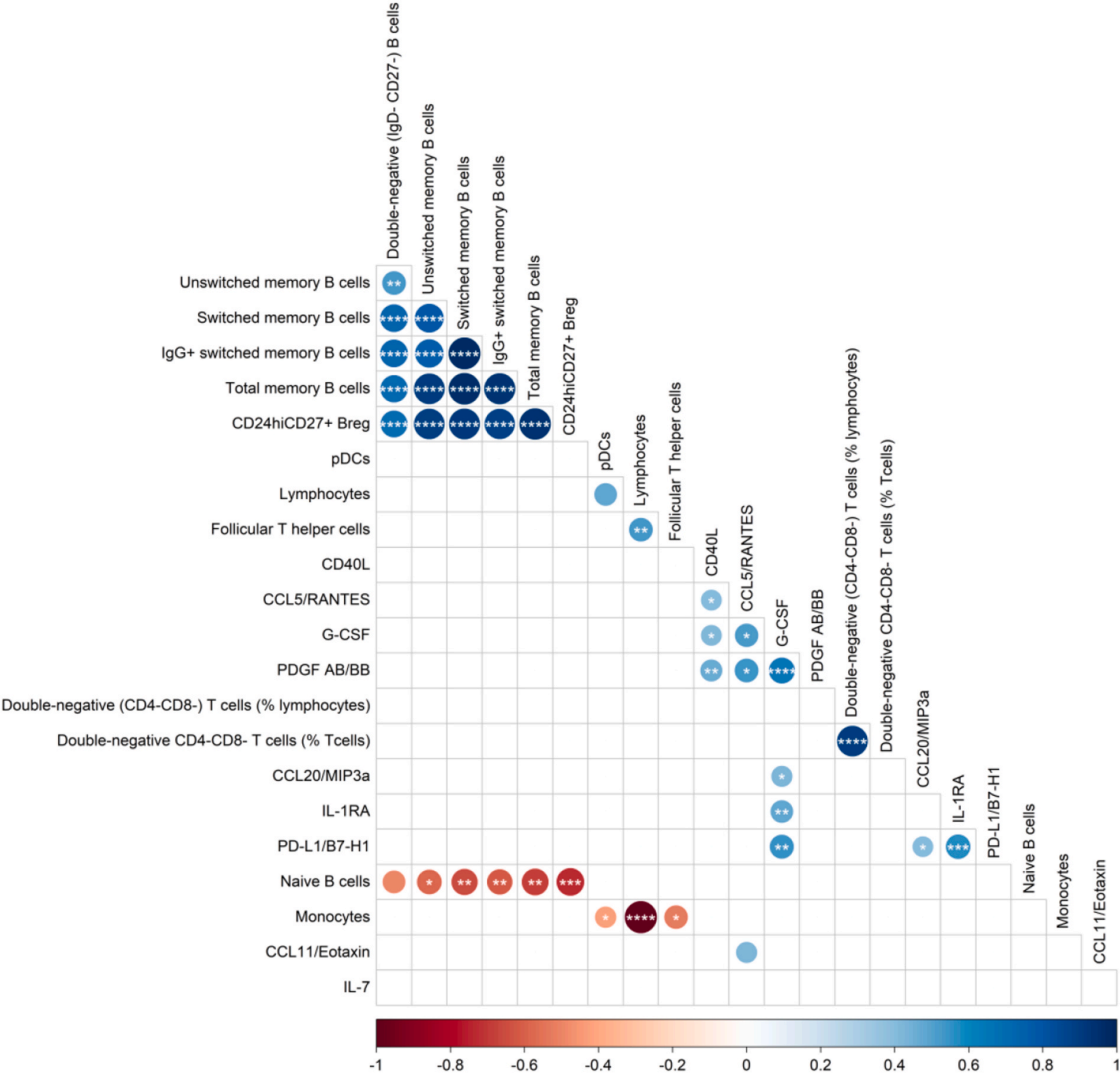
**Interparameter correlation between the immunological findings.** Heatmap demonstrating the correlations in pemphigus vulgaris (PV) patients between the significantly altered immune cell frequencies within the CD45^+^ compartment (monocytes, lymphocytes, plasmacytoid dendritic cells), subset frequencies within their respective parent populations (percent of lymphocytes: double-negative (CD4^−^CD8^−^) T cells; percent of T cells: double-negative (CD4^−^CD8^−^) T cells, follicular helper T cells; percent of B cells: naive B cells, double negative (IgD^−^CD27^−^) B cells, unswitched memory B cells, switched memory B cells, IgG^+^ switched memory B cells, total memory B cells, CD24^hi^CD27^+^ regulatory B cells), and serum analyte concentrations. The heatmap was generated using Ward's agglomerative hierarchical clustering. Circle size is proportional to the Spearman correlation coefficient, and correlations between −0.4 and 0.4 are not displayed. p-values were adjusted for multiple comparisons using the Benjamini-Hochberg false discovery rate correction. Full correlation statistics are provided in Supplementary File 2. *P < 0.05, **P < 0.01, ***P < 0.001, ****P < 0.0001. Abbreviations: Breg, regulatory B cell; PV, pemphigus vulgaris; nPV, rituximab-naive PV; nnPV, rituximab-non-naive PV; pDC, plasmacytoid dendritic cell; M, months.

To further examine relationships between significantly altered immune cell subsets and circulating cytokines/chemokines in PV, inter-parameter correlation analyses were performed. No significant correlations were observed between immune cell subset frequencies and circulating cytokine/chemokine levels measured by the Luminex assay. In contrast, correlations were detected within the cellular and cytokine/chemokine compartment (Fig. 7, Supplementary Fig. 8, and Supplementary File 2).

Within the B-cell compartment, non-naive B-cell subset frequencies showed moderate to strong positive correlations with each other, while naive B-cell frequency correlated negatively with the other significantly altered B-cell subsets. This indicates an inverse relationship between naive and more mature B-cell subsets in circulation.

Additional correlations were observed between myeloid and lymphoid populations within the CD45^+^ compartment. The pDC frequency correlated negatively with the monocyte frequency (r = −0.43).

Among the cytokines and chemokines, levels of IL-1RA, PD-L1/B7-H1/CD274, G-CSF, CCL5/RANTES, PDGF-AB/BB, CD40L, CCL11/eotaxin, and CCL20/MIP-3α displayed positive correlations with each other.

Together, these findings demonstrate coordinated relationships within B-cell subsets, between lymphoid and myeloid cell frequencies, and between circulating cytokines and chemokines. No associations were observed between cellular and soluble immune compartments in this analysis.

## 4 Discussion

In this exploratory study, we used spectral flow cytometry and multiplex Luminex analysis to characterize circulating immune cell and cytokine/chemokine profiles in active PV and to assess the effects of prior rituximab exposure. PV was characterized by shifts in monocyte and T-cell subsets, and increased levels of circulating inflammatory cytokines and chemokines. In parallel, subgroup analysis within the PV cohort showed that prior rituximab exposure was associated with sustained remodelling confined to the B-cell compartment.

Spectral flow cytometry analysis in active PV revealed a redistribution of *major immune cell populations* within the CD45^+^ PBMC compartment, characterized by increased monocyte frequency and reduced lymphocyte and pDC frequencies compared with HCs. Within the monocyte compartment, the *main* monocyte *subsets* showed a trend toward a classical monocyte-dominated profile in active PV. Monocytes have received relatively limited attention in the context of PV pathogenesis, and the distribution of *main* monocyte *subsets* in the circulation has not yet been characterized. Monocytes are recognized contributors to autoimmune disease through their roles in antigen presentation, cytokine production, and regulation of T- and B-cell responses [31,32]. Altered distributions of classical, intermediate, and non-classical monocytes have been reported across multiple autoimmune diseases in a disease- and context-dependent manner [32]. In PV, circulating monocytes in untreated patients exhibit a functionally pro-inflammatory transcriptional profile that is attenuated following treatment [31]. Beyond PV, accumulating evidence indicates that monocytes display substantial phenotypic and functional heterogeneity beyond the subsets captured in this study [26,33]. The findings underscore the need for high-dimensional and functional analyses to define how monocyte heterogeneity contributes to PV pathogenesis.

Within the lymphocyte compartment, the frequency of the *major* T-cell *population* was unchanged; however, alterations were observed in the *main* T-cell *subsets* in PV patients. Reduced frequencies of double-negative T cells and Tfh cells were observed in PV compared with HCs, while unsupervised UMAP analysis did not indicate broad restructuring of the T-cell landscape. Although data on double-negative T cells in PV are limited, altered frequencies of this population have been reported across several autoimmune diseases and are linked to inflammatory immune responses [34]. In contrast to previous reports describing increased Tfh frequencies in PV [35,36], a reduced Tfh frequency was observed in this study. This reduction may reflect immunosuppression-related effects, as Tfh frequencies showed a modest negative correlation with prednisone dosage, consistent with the known effects of corticosteroids on circulating Tfh cells [37]. In addition to alterations within the adaptive immune compartment, changes were also observed in innate immune populations, with a reduced pDC frequency in PV patients. However, circulating pDCs are also highly sensitive to glucocorticoids, and even low-dose prednisone can cause substantial depletion [38]. Consistent with this, in the present study, pDC frequency in PV patients showed a negative correlation with prednisone dosage. Together, these findings highlight the need to distinguish disease-related from corticosteroid-related immune alterations when interpreting circulating immune profiles in PV. A subgroup analysis restricted to PV patients not receiving immunosuppressive medication at the time of sampling showed a directionally similar pattern, with increased monocyte and decreased lymphocyte frequencies compared with HCs, although the pDC difference was less pronounced and the analysis should be interpreted cautiously due to the reduced sample size. These results support a shift toward an innate-dominated inflammatory profile during active disease, while also indicating that corticosteroid exposure may contribute to specific alterations, particularly reduced pDC and Tfh frequencies.

To define immune features associated with prior rituximab exposure during active disease, we compared nPV with nnPV patients. As expected, the most pronounced differences were confined to the B-cell compartment. The nnPV patients showed a shift toward a naive-dominant B-cell profile, suggesting sustained remodelling of the B-cell population even after absolute *major* B-cell *population* repopulation. This pattern is consistent with post-rituximab B-cell reconstitution, in which naive B cells recover earlier than memory and regulatory subsets [9,10]. This is further supported by correlation analyses showing that disease duration and time since the last rituximab administration, predominantly driven by nnPV patients, were negatively associated with unswitched memory and regulatory B-cell subset frequencies. Additionally, the distribution of non–B-cell *main immune cell subsets* was similar between nPV and nnPV patients, supporting that the long-term immunological effect of rituximab in this cohort was restricted to B-cell subset composition rather than reflecting broad remodelling across circulating immune lineages. Notably, in rheumatoid arthritis, differential recovery of memory B-cell subsets after rituximab has been shown to associate with treatment response and relapse risk [39]. In the present PV cohort, B-cell composition was assessed in patients with active disease following relapse and therefore does not capture immune profiles associated with sustained remission. Whether distinct patterns of B-cell reconstitution distinguish relapse from sustained remission in PV remains unknown and warrants further investigation across treatment response states.

Analysis of circulating cytokines and chemokines showed increased serum levels in PV patients compared with HCs. These included CD40L, IL-1RA, IL-7, CCL11/eotaxin, and CCL5/RANTES, previously reported to be elevated in active PV [[40], [41], [42], [43]], as well as G-CSF, CCL20/MIP-3α, PD-L1/B7-H1/CD274, and PDGF-AB/BB, which have not been described in PV before. Cytokine and chemokine concentrations did not differ between nPV and nnPV patients, indicating that there is no effect of prior rituximab exposure. However, for nnPV patients, time since last rituximab administration correlated negatively with IL-1RA, IL-7, and PD-L1/B7-H1/CD274, suggesting time-dependent variations in these soluble mediators during the post-rituximab period despite comparable overall distributions between the PV subgroups. Consistent with this, reduced IL-7 levels have previously been reported following rituximab treatment [44], whereas comparable post-rituximab associations for IL-1RA and PD-L1/B7-H1/CD274 have not been described. Together, these findings indicate that while the circulating cytokine profile in PV is primarily driven by active disease, IL-1RA, IL-7, and PD-L1/B7-H1/CD274 vary as a function of time since rituximab treatment.

Clinical correlation analyses indicated that corticosteroids may be a contributor to the observed increased frequency in memory and regulatory B cells, CCL11/eotaxin, and IL-7, and decreased frequency of pDCs and Tfh cells. Glucocorticoid treatment is associated with an increased frequency of memory B cells [45], supporting that systemic glucocorticoids can influence circulating B-cell subset distributions independently of B-cell–depleting therapy. Evidence regarding the effects of systemic corticosteroids on circulating CCL11/eotaxin and IL-7 levels remains limited. Circulating CCL11/eotaxin has been reported to remain unchanged following systemic corticosteroid treatment [46], suggesting that the positive association observed here may reflect PV-specific immune regulation rather than a direct corticosteroid effect. Given the role of CCL11/eotaxin in myeloid cell recruitment and activation, its association with prednisone dose is notable in the context of the monocyte alterations observed in PV and warrants further investigation. Similarly, although IL-7 is essential for the development, survival, and proliferation of lymphocytes, particularly T cells, the relationship between systemic corticosteroid exposure and circulating IL-7 remains poorly defined. Together, these findings underscore that corticosteroid exposure can shape B cell composition, pDCs, Tfh cells, and possibly also CCL11/eotaxin and IL7 in PV. Therefore, this should be considered when interpreting immune monitoring, post-rituximab B-cell reconstitution, and relapse-associated immune profiles.

Several limitations of this study should be acknowledged. First, the single-centre, cross-sectional design and small sample size may restrict the generalizability of the findings and prevent assessment of immune changes across treatment, remission, and relapse. Second, because many patients received immunosuppressive therapy, such as glucocorticoids, as part of standard clinical care, treatment-related immune modulation cannot be excluded and may contribute to the observed immune profiles in PV. Third, the inclusion of both rituximab-naive and rituximab-experienced relapsed patients introduces clinical heterogeneity that may influence immune findings. Fourth, the use of PBMCs limits the assessment of tissue-resident immune responses and excludes granulocyte populations, which may be relevant to PV pathogenesis and may contribute to relapse despite rituximab-mediated peripheral B-cell depletion. Finally, although the OMIP-069 panel provides broad immunophenotypic coverage, it limits the depth of characterization of rare antigen-specific T and B cells, and of other functional immune cell subsets. Future studies incorporating longitudinal sampling, sustained remission patients, tissue profiling, and higher-resolution functional analyses, including DSG3-tetramers, are necessary to further define the immune mechanisms underlying PV pathogenesis and the effects of rituximab.

In summary, our data support that active PV is associated with changes in circulating immune cell composition and cytokines/chemokines, while prior rituximab exposure in relapsed patients is linked to sustained remodelling confined to B-cell subset composition. By placing B-cell reconstitution in the context of broader circulating immune profiles during active disease, these results provide a framework for future longitudinal and tissue-integrated studies aimed at refining treatment strategies and immune monitoring beyond B-cells.

## Ethics statement

The study protocol was approved by the Institutional Review Board of the University Medical Center Groningen (METc no. 2012/151 and 2020/007). The study was conducted in accordance with applicable institutional and national regulations and with the principles of the Declaration of Helsinki. Written informed consent was obtained from all participants.

## Declaration of AI-assisted technologies in the manuscript preparation process

During the preparation of this work, the author(s) used ChatGPT (OpenAI) in order to assist with language and clarity of the text. After using this tool/service, the author(s) reviewed and edited the content as needed and take(s) full responsibility for the content of the published article.

## Funding statement

This work was supported by the De Cock–Hadders Foundation (grant number 2025-42), the Netherlands, and the Graduate School of Medical Sciences (GSMS), University of Groningen, the Netherlands.

## CRediT authorship contribution statement

**Anne-Lise Strandmoe:** Conceptualization, Data curation, Formal analysis, Funding acquisition, Methodology, Project administration, Software, Validation, Visualization, Writing – original draft, Writing – review & editing. **Carlo G. Bonasia:** Methodology, Software, Validation, Visualization, Writing – review & editing. **Nanthicha Inrueangsri:** Formal analysis, Methodology, Software, Validation, Visualization, Writing – review & editing. **Wayel H. Abdulahad:** Methodology, Writing – review & editing. **Inge M. Strating:** Investigation, Writing – review & editing. **Kevin P. Mennega:** Investigation, Writing – review & editing. **Theo Bijma:** Methodology, Writing – review & editing. **Gilles F.H. Diercks:** Supervision, Writing – review & editing. **Jeroen Bremer:** Supervision, Writing – review & editing. **Jon D. Laman:** Funding acquisition, Supervision, Writing – review & editing. **Barbara Horváth:** Supervision, Writing – review & editing. **Peter Heeringa:** Supervision, Writing – review & editing.

## Declaration of competing interest

The authors declare the following financial interests/personal relationships which may be considered as potential competing interests: AS, CGB, NI, WHA, IMS, KPM, GFHD, JB, JDL, and PH declare that they have no competing financial interests or personal relationships that could have appeared to influence the work reported in this paper. BH reports fees from Janssen-Cilag for advisory boards, educational grants, consultations, and investigator-initiated studies; AbbVie for advisory boards, educational grants, consultations, and investigator-initiated studies; Novartis Pharma for advisory boards, consultations, and investigator-initiated studies; UCB Pharma for advisory boards and consultations; Leo Pharma for consultations; Solenne B.V. for investigator-initiated studies; Celgene for consultations and investigator-initiated studies; Akari Therapeutics for consultations and investigator-initiated studies; Philips, Roche, Regeneron, and Sanofi for consultations; Argenx for advisory boards and consultations; and Boehringer Ingelheim for advisory boards and consultations. All contracts were reviewed by the Board of Directors of the UMCG, and all fees were paid to the institution.

## Data Availability

Data will be made available on request.

## References

[1] Kasperkiewicz M., Ellebrecht C.T., Takahashi H., Yamagami J., Zillikens D., Payne A.S., Amagai M. (2017). Pemphigus. Nat. Rev. Dis. Primers.

[2] Schmidt E., Kasperkiewicz M., Joly P. (2019). Pemphigus. Lancet.

[3] Ellebrecht C.T., Maseda D., Payne A.S. (2022). Pemphigus and pemphigoid: from disease mechanisms to druggable pathways. J. Invest. Dermatol..

[4] Hammers C.M., Stanley J.R. (2016). Mechanisms of disease: pemphigus and bullous pemphigoid. Annu. Rev. Pathol..

[5] Anhalt G.J., Labib R.S., Voorhees J.J., Beals T.F., Diaz L.A. (1982). Induction of pemphigus in neonatal mice by passive transfer of IgG from patients with the disease. N. Engl. J. Med..

[6] Hammers C.M., Chen J., Lin C., Kacir S., Siegel D.L., Payne A.S., Stanley J.R. (2015). Persistence of anti-desmoglein 3 IgG+ B-cell clones in pemphigus patients over years. J. Invest. Dermatol..

[7] Abrikosova V.A., Mokrushina Y.A., Ovchinnikova L.A., Larina E.N., Terekhov S.S., Baranova M.N., Lomakin Y.A., Balabashin D.S., Bobik T.V., Kaliberda E.N., Knorre V.D., Shpilevaya M.V., Aliev T.T., Deryabin D.G., Karamova A.E., Kubanov A.A., Kirpichnikov M.P., Smirnov I.V. (2023). B cell profiling in patients with Pemphigus vulgaris. Acta Nat..

[8] Pollmann R., Walter E., Schmidt T., Waschke J., Hertl M., Möbs C., Eming R. (2019). Identification of autoreactive B cell subpopulations in peripheral blood of autoimmune patients with Pemphigus vulgaris. Front. Immunol..

[9] Mouquet H., Musette P., Gougeon M.-L., Jacquot S., Lemercier B., Lim A., Gilbert D., Dutot I., Roujeau J.C., D'Incan M., Bedane C., Tron F., Joly P. (2008). B-cell depletion immunotherapy in pemphigus: effects on cellular and humoral immune responses. J. Invest. Dermatol..

[10] Boch K., Langan E.A., Schmidt E., Zillikens D., Ludwig R.J., Bieber K., Hammers C.M. (2022). Sustained CD19+CD27+ memory B cell depletion after rituximab treatment in patients with Pemphigus vulgaris. Acta Derm. Venereol..

[11] Tedbirt B., Maho-Vaillant M., Houivet E., Mignard C., Golinski M.-L., Calbo S., Prost-Squarcioni C., Labeille B., Picard-Dahan C., Chaby G., Richard M.-A., Tancrede-Bohin E., Duvert-Lehembre S., Delaporte E., Bernard P., Caux F., Alexandre M., Musette P., Ingen-Housz-Oro S., Vabres P., Quereux G., Dupuy A., Debarbieux S., Avenel-Audran M., D'Incan M., Bédane C., Bénéton N., Jullien D., Dupin N., Misery L., Machet L., Beylot-Barry M., Dereure O., Sassolas B., Benichou J., Joly P., Hébert V. (2024). Sustained remission without corticosteroids among patients with pemphigus who had rituximab as first-line therapy. Journal of the American Medical Association Dermatology.

[12] Polakova A., Kauter L., Ismagambetova A., Didona D., Solimani F., Ghoreschi K., Hertl M., Möbs C., Hudemann C. (2022). Detection of rare autoreactive T cell subsets in patients with Pemphigus vulgaris. Front. Immunol..

[13] Holstein J., Solimani F., Baum C., Meier K., Pollmann R., Didona D., Tekath T., Dugas M., Casadei N., Hudemann C., Polakova A., Matthes J., Schäfer I., Yazdi A.S., Eming R., Hertl M., Pfützner W., Ghoreschi K., Möbs C. (2021). Immunophenotyping in pemphigus reveals a Th17/Tfh17 cell–dominated immune response promoting desmoglein1/3-specific autoantibody production. J. Allergy Clin. Immunol..

[14] Kim A.R., Han D., Choi J.Y., Seok J., Kim S.-E., Seo S.-H., Takahashi H., Amagai M., Park S.-H., Kim S.-C., Shin E.-C., Kim J.H. (2020). Targeting inducible costimulator expressed on CXCR5+PD-1+ Th cells suppresses the progression of Pemphigus vulgaris. J. Allergy Clin. Immunol..

[15] Han K., Li S.-S., Pan W., Xu M.-N., Zhong M.-Z., Zhang W.-J., Huang X.-W., Zeng K. (2023). ERK/MEK pathway regulates Th17 cell differentiation in patients with Pemphigus vulgaris. Indian J. Dermatol..

[16] Asothai R., Anand V., Das D., Antil P.S., Khandpur S., Sharma V.K., Sharma A. (2015). Distinctive treg associated CCR4-CCL22 expression profile with altered frequency of Th17/Treg cell in the immunopathogenesis of Pemphigus vulgaris. Immunobiology.

[17] Rizzo C., Fotino M., Zhang Y., Chow S., Spizuoco A., Sinha A.A. (2005). Direct characterization of human T cells in Pemphigus vulgaris reveals elevated autoantigen-specific Th2 activity in association with active disease. Clin. Exp. Dermatol..

[18] Akman Karakaş A., Ergün E., Sayın Ekinci N., Toylu A., Uzun S., Alpsoy E. (2023). The role of T follicular helper cells in clinical remission and relapse in patients with pemphigus treated with rituximab. Acta Dermatovenerol. Croat..

[19] Hébert V., Novarino J., Maho-Vaillant M., Perals C., Calbo S., Golinski M.-L., Martinez F., Joly P., Fazilleau N. (2024). The emergence of circulating activated autoreactive desmoglein 3-specific follicular regulatory T cells is associated with long-term efficacy of rituximab in patients with Pemphigus vulgaris. Br. J. Dermatol..

[20] Zhu Y., Su J., Zhang P., Deng M., Wu R., Liu Y., Su Y., Li S. (2023). The dysregulation of circulating innate lymphoid cells is related to autoantibodies in Pemphigus vulgaris. Int. Immunopharmacol..

[21] Hooda V., Khandpur S., Sharma A. (2025). Augmented IFNγ producing ILC1 and IL 17 producing ILC3 in pemphigus vulgaris: plausible therapeutic target. Cell. Immunol..

[22] Hooda V., Khandpur S., Arava S., Sharma A. (2024). Distorted frequency and functionality of natural killer cells in pemphigus vulgaris: a potential therapeutic target. Immunol. Lett..

[23] Das D., Singh A., Antil P.S., Sharma D., Arava S., Khandpur S., Sharma A. (2020). Distorted frequency of dendritic cells and their associated stimulatory and inhibitory markers augment the pathogenesis of Pemphigus vulgaris. Immunol. Res..

[24] Sharma A., Chauhan A., Mehta H., Handa S., Sachdeva N., Mahajan R., Pal A., De D. (2025). Influence of CD16 (FcγRIII) expression on peripheral blood mononuclear cells on response to treatment with rituximab in pemphigus: a cross-sectional study. Indian Dermatol. Online J..

[25] Murrell D.F., Peña S., Joly P., Marinovic B., Hashimoto T., Diaz L.A., Sinha A.A., Payne A.S., Daneshpazhooh M., Eming R., Jonkman M.F., Mimouni D., Borradori L., Kim S.-C., Yamagami J., Lehman J.S., Saleh M.A., Culton D.A., Czernik A., Zone J.J., Fivenson D., Ujiie H., Wozniak K., Akman-Karakaş A., Bernard P., Korman N.J., Caux F., Drenovska K., Prost-Squarcioni C., Vassileva S., Feldman R.J., Cardones A.R., Bauer J., Ioannides D., Jedlickova H., Palisson F., Patsatsi A., Uzun S., Yayli S., Zillikens D., Amagai M., Hertl M., Schmidt E., Aoki V., Grando S.A., Shimizu H., Baum S., Cianchini G., Feliciani C., Iranzo P., Mascaró J.M., Kowalewski C., Hall R., Groves R., Harman K.E., Marinkovich M.P., Maverakis E., Werth V.P. (2020). Diagnosis and management of pemphigus: recommendations of an international panel of experts. J. Am. Acad. Dermatol..

[26] Bonasia C.G., Inrueangsri N., Bijma T., Mennega K.P., Wilbrink R., Arends S., Abdulahad W.H., Bos N.A., Rutgers A., Heeringa P. (2024). Circulating immune profile in granulomatosis with polyangiitis reveals distinct patterns related to disease activity. J. Autoimmun..

[27] Park L.M., Lannigan J., Jaimes M.C. (2020). OMIP‐069: forty‐color full spectrum flow cytometry panel for deep immunophenotyping of major cell subsets in human peripheral blood. Cytometry A.

[28] Szabo P.A., Levitin H.M., Miron M., Snyder M.E., Senda T., Yuan J., Cheng Y.L., Bush E.C., Dogra P., Thapa P., Farber D.L., Sims P.A. (2019). Single-cell transcriptomics of human T cells reveals tissue and activation signatures in health and disease. Nat. Commun..

[29] Mathew D., Giles J.R., Baxter A.E., Oldridge D.A., Greenplate A.R., Wu J.E., Alanio C., Kuri-Cervantes L., Pampena M.B., D'Andrea K., Manne S., Chen Z., Huang Y.J., Reilly J.P., Weisman A.R., Ittner C.A.G., Kuthuru O., Dougherty J., Nzingha K., Han N., Kim J., Pattekar A., Goodwin E.C., Anderson E.M., Weirick M.E., Gouma S., Arevalo C.P., Bolton M.J., Chen F., Lacey S.F., Ramage H., Cherry S., Hensley S.E., Apostolidis S.A., Huang A.C., Vella L.A., Betts M.R., Meyer N.J., Wherry E.J., Alam Z., Addison M.M., Byrne K.T., Chandra A., Descamps H.C., Kaminskiy Y., Hamilton J.T., Noll J.H., Omran D.K., Perkey E., Prager E.M., Pueschl D., Shah J.B., Shilan J.S., Vanderbeck A.N. (2020). Deep immune profiling of COVID-19 patients reveals distinct immunotypes with therapeutic implications. Science.

[30] Saris A., Reijnders T.D.Y., Nossent E.J., Schuurman A.R., Verhoeff J., van Asten S., Bontkes H., Blok S., Duitman J., Bogaard H.-J., Heunks L., Lutter R., van der Poll T., Garcia Vallejo J.J. (2021). Distinct cellular immune profiles in the airways and blood of critically ill patients with COVID-19. Thorax.

[31] Duan S., Li Q., Wang F., Kuang W., Dong Y., Liu D., Wang J., Li W., Chen Q., Zeng X., Li T. (2024). Single-cell transcriptomes and immune repertoires reveal the cell state and molecular changes in Pemphigus vulgaris. J. Immunol..

[32] Kawakami A., Iwamoto N., Fujio K. (2022). The role of monocytes/macrophages in autoimmunity and autoinflammation. Front. Immunol..

[33] V Ruder A., Wetzels S.M.W., Temmerman L., Biessen E.A.L., Goossens P. (2023). Monocyte heterogeneity in cardiovascular disease. Cardiovasc. Res..

[34] Li H., Tsokos G.C. (2021). Double-negative T cells in autoimmune diseases. Curr. Opin. Rheumatol..

[35] Hennerici T., Pollmann R., Schmidt T., Seipelt M., Tackenberg B., Möbs C., Ghoreschi K., Hertl M., Eming R. (2016). Increased frequency of T follicular helper cells and elevated interleukin-27 plasma levels in patients with pemphigus. Public Library of Science ONE.

[36] Fang H., Li Q., Wang G. (2020). The role of T cells in Pemphigus vulgaris and bullous pemphigoid. Autoimmun. Rev..

[37] Liu Y., Zhao P., Qu Z., Ayana D.A., Jiang Y. (2014). Frequency of CD4+CXCR5+ Tfh cells in patients with hepatitis b virus-associated membranous nephropathy. Int. Immunopharmacol..

[38] Shodell M., Shah K., Siegal F.P. (2003). Circulating human plasmacytoid dendritic cells are highly sensitive to corticosteroid administration. Lupus.

[39] Roll P., Dörner T., Tony H. (2008). Anti‐CD20 therapy in patients with rheumatoid arthritis: predictors of response and B cell subset regeneration after repeated treatment. Arthritis Rheum..

[40] Caproni M., Antiga E., Torchia D., Volpi W., del Bianco E., Cappetti A., Feliciani C., Fabbri P. (2007). The CD40/CD40 ligand system is involved in the pathogenesis of pemphigus. Clin. Immunol..

[41] You S., Ouyang J., Wu Q., Zhang Y., Gao J., Luo X., Wang Y., Wu Y., Jiang F. (2024). Comparison of serum cytokines and chemokines levels and clinical significance in patients with pemphigus vulgaris—A retrospective study. Exp. Dermatol..

[42] Schwartz R.R., Seiffert-Sinha K., Sinha A.A. (2024). Cytokine profiling reveals HLA-linked Th2 and Th17 driven immune activation in Pemphigus vulgaris patients and genetically susceptible healthy controls. Front. Immunol..

[43] Timoteo R.P., da Silva M.V., Miguel C.B., Silva D.A.A., Catarino J.D.S., Rodrigues Junior V., Sales-Campos H., Freire Oliveira C.J. (2017). Th1/Th17-related cytokines and chemokines and their implications in the pathogenesis of Pemphigus vulgaris. Mediat. Inflamm..

[44] Olusi O. (2012). Effects of rituximab treatment on the serum concentrations of vitamin D and interleukins 2, 6, 7, and 10 in patients with rheumatoid arthritis. Biologics.

[45] Lanzillotta M., Della-Torre E., Milani R., Bozzolo E., Bozzalla-Cassione E., Rovati L., Arcidiacono P.G., Partelli S., Falconi M., Ciceri F., Dagna L. (2019). Effects of glucocorticoids on B-cell subpopulations in patients with IgG4-related disease. Clin. Exp. Rheumatol..

[46] Tateno H., Nakamura H., Minematsu N., Nakajima T., Takahashi S., Nakamura M., Fukunaga K., Asano K., Lilly C.M., Yamaguchi K. (2004). Plasma eotaxin level and severity of asthma treated with corticosteroid. Respir. Med..

