# Supplementary material for: Rituximab remodels the circulating immune landscape in pemphigus vulgaris revealed by high-dimensional immune profiling
Source: J Transl Autoimmun. 2026 Aug 3;13:100390. doi: 10.1016/j.jtauto.2026.100390 (PMC13475701; doi:10.1016/j.jtauto.2026.100390)
Supplement: Supplementary file 1 [file mmc1.docx]

**Supplementary figures**

**Supplementary Figure 1 – Gating strategy of the spectral flow cytometry OMIP-069 panel** with adaptations from Bonasia et al (10.1016/j.jaut.2024.103236). Abbreviations: NK, Natural Killer; ILC, Innate Lymphoid Cell.

**Supplementary Figure 2** – **Violin plots showing the frequencies of all measured major immune cell populations within the CD45⁺ compartment, and basophil frequency within the live cell population.** Comparisons were performed between healthy controls and the pemphigus vulgaris cohort, as well as between rituximab-naive and rituximab-non-naive patients. Mann-Whitney U tests were used for all group comparisons, and p-values were adjusted for multiple comparisons using the Benjamini-Hochberg false discovery rate correction. Significant differences (adjusted P < 0.05) are indicated on the plots.

Abbreviations: NK, Natural Killer; ILC, Innate Lymphoid Cell; pDC, plasmacytoid Dendritic Cell; PV, Pemphigus Vulgaris; nPV, rituximab-naive PV; nnPV, rituximab-non-naive PV; HC, Healthy Control.

**Supplementary Figure 3** – **Violin plots showing the frequencies of all measured main immune cell subsets within their respective major parent populations**, including lymphocytes, monocytes, NK cells, ILCs, dendritic cells, T cells, and B cells. Comparisons were performed between healthy controls and the pemphigus vulgaris cohort, as well as between rituximab-naive and rituximab-non-naive patients. Mann-Whitney U tests were used for all group comparisons, and p-values were adjusted for multiple comparisons using the Benjamini-Hochberg false discovery rate correction. Significant differences (adjusted P < 0.05) are indicated on the plots. Abbreviations: NK, natural killer; ILC, innate lymphoid cell; pDC, plasmacytoid dendritic cell; PV, pemphigus vulgaris; nPV, rituximab-naive PV; nnPV, rituximab-non-naive PV; HC, healthy control; gd, γδ; Treg, regulatory T cell; TEMRA, terminally differentiated effector memory CD45RA^+^; Breg, regulatory B cell.

**a.**

**b.**

**Supplementary Figure 4 – Frequencies of manually gated major immune cell populations in PV patients not receiving systemic immunosuppressive treatment.**

**a.** Heatmap of the frequencies of manually gated major populations within the CD45⁺ cell population and basophil frequency within the live-cell population in pemphigus vulgaris (PV) patients not receiving systemic immunosuppressive treatment at the time of sampling, including prednisone or azathioprine (n = 14: nnPV, n = 10; nPV, n = 4), and healthy controls (HCs, n = 24). Ward’s hierarchical agglomerative clustering was applied to generate the heatmap. Cell frequencies were transformed into z-scores, with the blue-red colour scale representing low to high frequency, respectively.

**b.** Violin plots showing the frequencies of manually gated major immune-cell populations within the CD45⁺ compartment in the same subgroup of PV patients and HCs. Mann-Whitney U tests were used, and p-values were adjusted for multiple comparisons using the Benjamini-Hochberg false discovery rate correction. Significant differences (adjusted P < 0.05) are indicated in the plots.

**Supplementary Figure 5 – Violin plots showing the absolute counts of all measured major immune cell populations** in rituximab-naive (nPV) and rituximab-non-naive (nnPV) patients. Absolute values were compared using Mann-Whitney U tests, and p-values were adjusted for multiple comparisons using the Benjamini-Hochberg false discovery rate correction. No significant differences were observed between nPV and nnPV patients. Abbreviations: PV, pemphigus vulgaris; nPV, rituximab-naive PV; nnPV, rituximab-non-naive PV; ILC, innate lymphoid cell; NK, natural killer; pDC, plasmacytoid dendritic cell; TCRgd, T-cell receptor γδ.

**Supplementary Figure 6** – **Violin plots showing the concentrations of all 25 measured serum analytes using the Human XL Cytokine Luminex assay.** Comparisons were performed between healthy controls (HCs) and the pemphigus vulgaris (PV) cohort, as well as between rituximab-naive (nPV) and rituximab-non-naive (nnPV) patients. Mann-Whitney U tests were used for all group comparisons, and p-values were adjusted for multiple comparisons using the Benjamini-Hochberg false discovery rate correction. Significant differences (adjusted P < 0.05) are indicated on the relevant plots. Abbreviations: PV, pemphigus vulgaris; nPV, rituximab-naive PV; nnPV, rituximab-non-naive PV; HC, healthy control.

**Supplementary Figure 7** – **Correlations between significantly altered immune cell frequencies and clinical parameters in pemphigus vulgaris patients.** Included immune parameters: immune cell frequencies within the CD45⁺ compartment (monocytes, lymphocytes, plasmacytoid dendritic cells), subset frequencies within major immune populations (percent of lymphocytes: double negative (CD4^-^CD8^-^) T cells; percent of T cells: double negative (CD4^-^CD8^-^) T cells, follicular helper T cells; percent of B cells: naive B cells, double negative (IgD^-^CD27^-^) B cells, unswitched memory B cells, switched memory B cells, IgG⁺ switched memory B cells, total memory B cells, CD24^hi^CD27⁺ regulatory B cells), and serum concentrations of analytes. Spearman correlation coefficients were calculated, and p-values were adjusted for multiple comparisons using the Benjamini-Hochberg false discovery rate correction. Full correlation statistics are provided in Supplementary File 1. Abbreviations: RTX, rituximab; DSG, desmoglein; Breg, regulatory B cell; PV, pemphigus vulgaris; nPV, rituximab-naive PV; nnPV, rituximab-non-naive PV; pDC, plasmacytoid dendritic cell; M, months.

**Supplementary Figure 8** – **Interparameter correlations between significantly altered immune parameters in pemphigus vulgaris patients.** Included immune parameters: immune cell frequencies within the CD45⁺ compartment (monocytes, lymphocytes, plasmacytoid dendritic cells), subset frequencies within major immune populations (percent of lymphocytes: double negative (CD4^-^CD8^-^) T cells; percent of T cells: double negative (CD4^-^CD8^-^) T cells, follicular helper T cells; percent of B cells: naive B cells, double negative (IgD^-^CD27^-^) B cells, unswitched memory B cells, switched memory B cells, IgG⁺ switched memory B cells, total memory B cells, CD24^hi^CD27⁺ regulatory B cells), and serum concentrations of analytes. Spearman correlation was used, and p-values were adjusted for multiple comparisons using the Benjamini-Hochberg false discovery rate correction. Full correlation statistics are provided in Supplementary File 2. Abbreviations: RTX, rituximab; DSG, desmoglein; Breg, regulatory B cell; PV, pemphigus vulgaris; nPV, rituximab-naive PV; nnPV, rituximab-non-naive PV; pDC, plasmacytoid dendritic cell.

**a.**

**b.**

**c.**

**d.**

**Supplementary Figure 9 – Unsupervised clustering of T cell subsets within the total T cell population. a**. UMAP of T cells with FlowSOM overlay generated from merged samples of healthy controls (HCs; 448,266 cells, n = 13), rituximab-naive PV patients (nPV; 375,972 cells, n = 13), and rituximab-non-naive PV patients (nnPV; 436,123 cells, n = 13). Samples were selected to match age, sex, and total cell numbers as closely as possible across groups. **b**. Marker expression profiles for the ten identified T-cell clusters, including UMAP with FlowSOM overlay and expression of CD4, CD8, CD45RA, CD28, CD27, CCR7, CD25, CD95, and CD127. **c**. Heatmap displaying the marker expression profile of each T-cell cluster using min-max scaling (white indicates minimum, pink indicates maximum). **d**. Violin plots showing the frequency (%) of each T-cell cluster within the total T-cell population for HCs, nPV, and nnPV patients.

Abbreviations: EM, effector memory; TEMRA, T effector memory CD45RA⁺; TCM, T central memory; Treg, regulatory T cell.
