# Supplementary material for: Rituximab remodels the circulating immune landscape in pemphigus vulgaris revealed by high-dimensional immune profiling
Source: J Transl Autoimmun. 2026 Aug 3;13:100390. doi: 10.1016/j.jtauto.2026.100390 (PMC13475701; doi:10.1016/j.jtauto.2026.100390)
Supplement: Supplementary file 2 [file mmc2.docx]

**Tables Supplementary**

**Supplementary Table 1 -** Forty-colour spectral flow cytometry Optimized Multicolor Immunofluorescence Panel-069. Antibodies and their respective specificity, fluorochrome, clone, supplier, and purpose. Abbreviations: NK, natural killer; Treg, regulatory T cell.

| **Specificity** | **Fluorochrome** | **Clone** | **Supplier** | **Volume (μL)** | **Purpose** |
| --- | --- | --- | --- | --- | --- |
| Viability | Live Dead UV Blue | - | Thermo Fisher Scientific | 0.125 | Viability |
| TCRγδ | PerCP-eFluor 710 | B1.1 | Thermo Fisher Scientific | 1.25 | Pan γδ T cell |
| CCR5 | BUV563 | 2D7/CCR5 | BD Biosciences | 2.5 | Chemokine receptor; Monocyte, dendritic cell, T cell, and B cell differentiation |
| CXCR5 | BV750 | RF8B2 | BD Biosciences | 1.25 | Chemokine receptor; T cell differentiation |
| IgG | BV605 | G18-145 | BD Biosciences | 5 | B-cell differentiation |
| CD8 | BUV805 | SK1 | BD Biosciences | 0.625 | CD8 T, NK, and NKT-Like cells |
| CD16 | BUV496 | 3G8 | BD Biosciences | 1.25 | Monocyte, NK cell, and dendritic cell differentiation |
| CD39 | BUV661 | TU66 | BD Biosciences | 5 | B cell, Tregs, and monocyte differentiation |
| IgD | BV480 | IA6-2 | BD Biosciences | 0.625 | B-cell differentiation |
| CD141 | BB515 | 1A4 | BD Biosciences | 1.25 | Dendritic cell differentiation |
| CD56 | BUV737 | NCAM16.2 | BD Biosciences | 0.156 | Pan NK cell, γδ T cell activation |
| CD27 | APC-H7 | M-T271 | BD Biosciences | 1.25 | T and B cell differentiation |
| CD45RA | BUV395 | 5H9 | BD Biosciences | 1.25 | T cell and dendritic cell differentiation |
| CD127 | APC-R700 | HIL-7R-M21 | BD Biosciences | 2.5 | Cytokine receptor; T cell differentiation |
| CD314 | BUV615 | 1D11 | BD Biosciences | 5 | NK cell differentiation |
| CD45 | PerCP | 2D1 | BioLegend | 2.5 | Leukocytes |
| CD3 | BV510 | SK7 | BioLegend | 4 | Pan T cell, NKT-Like cells |
| PD-1 | BV785 | EH12.2H7 | BioLegend | 5 | T cell inhibitory receptor |
| CD14 | Spark Blue 550 | 63D3 | BioLegend | 10 | Monocyte differentiation |
| CD19 | Spark NIR 685 | HIB19 | BioLegend | 1.25 | B cells |
| IgM | BV570 | MHM-88 | BioLegend | 3.125 | B-cell differentiation |
| CD1c | Alexa Fluor 647 | L161 | BioLegend | 2.5 | Dendritic cells, NKT-Like cells |
| CD2 | PerCP-Cy5.5 | TS1/8 | BioLegend | 2.5 | NK cell differentiation |
| CCR7 | BV421 | G043H7 | BioLegend | 7 | T cell differentiation |
| CD95 | PE-Cy5 | DX2 | BioLegend | 1.25 | T cell and B cell differentiation |
| CD337 | PE-Dazzle594 | P30-15 | BioLegend | 5 | NK cell differentiation |
| CCR6 | BV711 | G034E3 | BioLegend | 1.25 | Chemokine receptor; T cell and B cell differentiation |
| CXCR3 | PE-Cy7 | G025H7 | BioLegend | 10 | Chemokine receptor; Dendritic cell, T cell, and B cell differentiation |
| HLA-DR | PE-Fire810 | L243 | BioLegend | 5 | T cell and monocyte activation, NK cell lineage discrimination, dendritic cell lineage marker |
| CD38 | APC-Fire810 | HIT2 | BioLegend | 5 | Monocyte, dendritic cell, T cell, and B cell activation/differentiation |
| CD57 | FITC | HNK-1 | BioLegend | 2.5 | NK and CD8+ T cell immune senescence |
| CD28 | BV650 | CD28.2 | BioLegend | 2.5 | T cell and NK cell differentiation |
| CD25 | PE-Alexa Fluor700 | CD25-3G10 | Thermo Fisher Scientific | 5 | Regulatory T cells |
| CD11c | eFluor 450 | 3.9 | Thermo Fisher Scientific | 1.25 | Dendritic cell differentiation |
| CD20 | cFluor V547 | 2H7 | CYTEK | 5 | B cells |
| CD24 | PE-Alexa Fluor 610 | SN3 | Thermo Fisher Scientific | 5 | B-cell differentiation |
| CD123 | Super Bright 436 | 6H6 | Thermo Fisher Scientific | 0.625 | Plasmacytoid dendritic cells |
| CD159a | APC | REA110 | Miltenyi | 2 | NK, NKT-Like, and γδ T cell activation/differentiation |
| CD159c | PE | REA205 | Miltenyi | 2 | NK cell differentiation |
| CD4 | cFluor YG584 | SK3 | CYTEK | 0.5 | CD4 T and NKT-Like cells |

**Supplementary Table 2 -** The human XL cytokine Luminex performance panel, 46 analytes.

| **Analytes** | |
| --- | --- |
| CCL2/JE/MCP-1 | IL-2 |
| CCL3/MIP-1α | IL-3 |
| CCL4/MIP-1β | IL-4 |
| CCL5/RANTES | IL-5 |
| CCL11/Eotaxin | IL-6 |
| CCL19/MIP-3β | IL-7 |
| CCL20/MIP-3α | IL-8/CXCL8 |
| CD40 Ligand/TNFSF5 | IL-9 |
| CXCL1/GRO-α/KC/CINC-1 | IL-10 |
| CXCL2/GRO-β/MIP-2/CINC-3 | IL-12 p70 |
| CXCL10/IP-10/CRG-2 | IL-13 |
| EGF | IL-15 |
| FGF basic/FGF2/bFGF | IL-17/IL-17A |
| Flt-3 Ligand/FLT3L | IL-17E/IL-25 |
| G-CSF | IL-33 |
| GM-CSF | Lymphotoxin-α/TNF-β |
| Granzyme B | PD-L1/B7-H1/CD274 |
| IFN-α2/IFNA2 | PDGF-AA |
| IFN-β | PDGF-AB/BB |
| IFN-γ | TGF-α |
| IL-1-α/IL-1F1 | TNF-α |
| IL-1-β/IL-1F2 | TRAIL/TNFSF10 |
| IL-1RA/IL-1F3 | VEGF |

**Supplementary Table 3 -** Major immune cell populations and main immune cell subsets. Abbreviations: NK, natural killer; ILC, innate lymphoid cell; pDC, plasmacytoid dendritic cell; Breg, regulatory B cell.

| **Major immune cell population** | **Main immune cell subsets** |
| --- | --- |
| Basophils | — |
| Monocytes | Classical monocytes; intermediate monocytes; non-classical monocytes |
| Lymphocytes | Total T cells; total B cells; total NK cells; total ILCs |
| T cells | CD4⁺ T cells; CD8⁺ T cells; double-negative CD4⁻CD8⁻ T cells; double-positive CD4⁺CD8⁺ T cells; γδ T cells; NKT-like cells |
| B cells | Naive B cells; double-negative IgD⁻CD27⁻ B cells; transitional B cells; total memory B cells; unswitched memory B cells; switched memory B cells; IgG⁺ switched memory B cells; plasmablasts; CD24^hi^CD38^hi^ Breg; CD24^hi^CD27⁺ Breg |
| NK cells | Early NK cells; mature NK cells; terminal NK cells |
| Total ILCs | CD4⁺ ILCs; CD4⁻ ILCs |
| CD4⁺ T cells | Naive CD4⁺ T cells; central memory CD4⁺ T cells; effector memory CD4⁺ T cells; early-like effector memory CD4⁺ T cells; effector effector memory CD4⁺ T cells; terminal effector memory CD4⁺ T cells; regulatory T cells; CD39⁻CD45RA⁻ Treg; CD39⁻CD45RA⁺ Treg; CD39⁺CD45RA⁻ Treg; follicular T helper cells |
| CD8⁺ T cells | Naive CD8⁺ T cells; central memory CD8⁺ T cells; effector memory CD8⁺ T cells; early-like effector memory CD8⁺ T cells; effector effector memory CD8⁺ T cells; intermediate effector memory CD8⁺ T cells; terminal effector CD8⁺ T cells; CD8⁺ TEMRA |
| TCRγδ⁺ T cells | CCR7⁻CD45RA⁺⁺ γδ T cells; CCR7⁻CD45RA⁻ γδ T cells; CCR7⁺ γδ T cells |
| NKT-like cells | CD2⁺CD8⁺ NKT-like cells; CD2⁺CD8⁻ NKT-like cells; CD2⁻ NKT-like cells |
| pDCs / DCs | Total DCs (CD3⁻CD19⁻CD56⁻); CD11c⁺ DCs; CD123⁺ pDCs |

**Supplementary Table 4** – Frequency of major immune cell populations within the CD45⁺ cell compartment. Frequencies were calculated by dividing the count of each major immune cell population by the total CD45⁺ cell count. Basophil frequency was determined within the live cell population by calculating the percentage using the basophil (HLA-DR⁻CD38⁺) count and the live cell count. Comparisons were performed between healthy controls (HCs) and the total pemphigus vulgaris (PV) cohort, as well as between rituximab-naive (nPV) and rituximab-non-naive (nnPV) patients. Mann-Whitney U tests were used for all group comparisons, and p-values were adjusted for multiple testing using the Benjamini-Hochberg (BH) false discovery rate correction. Significant differences (adjusted P < 0.05) are indicated in bold. Abbreviations: NK, natural killer; ILC, innate lymphoid cell; pDC, plasmacytoid dendritic cell.

|  | **Frequency (%) within the CD45^+^ population** | | | **Mann-Whitney U Test** | | | |
| --- | --- | --- | --- | --- | --- | --- | --- |
|  |  |  |  | **HC vs PV** | | **nPV vs nnPV** | |
| **Population** | **HC**  **n=24** | **nPV**  **n=13** | **nnPV**  **n=14** | **p-value** | **BH**  **p-value** | **p-value** | **BH**  **p-value** |
| Basophils | 0.47 (0.01 - 1.63) | 0.22 (0.00 - 0.90) | 0.65 (0.00 - 2.20) | 0.7152 | 0.7802 | 0.0078 | 0.0936 |
| Monocytes | 14.90 (8.00 - 38.14) | 25.98 (11.10 - 50.29) | 27.13 (10.68 - 58.53) | 0.0002 | **0.0012** | 0.8995 | 0.9430 |
| Lymphocytes | 81.03 (58.91 - 89.91) | 70.76 (26.51 - 87.49) | 70.88 (39.04 - 87.50) | 0.0003 | **0.0012** | 0.9430 | 0.9430 |
| T cells | 57.35 (41.48 - 78.15) | 53.72 (21.98 - 78.83) | 51.62 (22.78 - 67.66) | 0.0438 | 0.0960 | 0.7203 | 0.9430 |
| B cells | 5.16 (1.16 - 10.76) | 3.98 (0.41 - 9.16) | 4.34 (0.55 - 12.20) | 0.1395 | 0.2093 | 0.7564 | 0.9430 |
| NK cells | 14.06 (2.47 - 34.21) | 9.88 (2.15 - 43.31) | 9.87 (2.89 - 14.93) | 0.0480 | 0.0960 | 0.5502 | 0.9430 |
| Total ILCs | 0.61 (0.25 - 1.26) | 0.46 (0.07 - 1.13) | 0.48 (0.17 - 1.07) | 0.2890 | 0.3853 | 0.8300 | 0.9430 |
| CD4^+^ T cells | 37.12 (17.55 - 58.34) | 28.65 (7.81 - 42.94) | 32.67 (10.48 - 50.63) | 0.0713 | 0.1222 | 0.1694 | 0.5082 |
| CD8^+^ T cells | 11.34 (4.73 - 36.48) | 8.41 (1.49 - 30.78) | 9.25 (4.03 - 24.72) | 0.3834 | 0.4601 | 0.9430 | 0.9430 |
| TCRδ**γ**^+^ T cells | 1.69 (0.56 - 7.82) | 1.59 (0.68 - 3.75) | 1.06 (0.53 - 3.26) | 0.0480 | 0.0960 | 0.0683 | 0.4098 |
| NKT-like cells | 4.46 (1.38 - 6.73) | 3.99 (0.80 - 14.79) | 3.93 (1.77 - 11.83) | 0.8006 | 0.8006 | 0.7564 | 0.9430 |
| pDCs | 0.08 (0.02 - 0.39) | 0.03 (0.00 - 0.17) | 0.04 (0.01 - 0.15) | 0.0002 | **0.0012** | 0.1694 | 0.5082 |

**Supplementary Table 5** – Frequency of main immune cell subsets within their respective major parent compartments. Frequencies were calculated by dividing the number of each main immune cell subset by the total number of cells in the corresponding major parent gate, representing the major immune population from which the subset was derived. Comparisons were performed between healthy controls (HCs) and the overall pemphigus vulgaris (PV) cohort, as well as between rituximab-naive (nPV) and rituximab-non-naive (nnPV) patients. Mann-Whitney U tests were used for all group comparisons, and p-values were adjusted for multiple testing using the Benjamini-Hochberg (BH) false discovery rate correction. Significant differences (adjusted P < 0.05) are indicated in bold. Abbreviations: NK, natural killer; ILC, innate lymphoid cell; pDC, plasmacytoid dendritic cell; Breg, regulatory B cell.

|  |  | **Frequency (%) in parent gate** | | | **Mann-Whitney U Test** | | | | |
| --- | --- | --- | --- | --- | --- | --- | --- | --- | --- |
|  |  |  |  |  | **HC vs PV** | | **nPV vs nnPV** | | |
| **Subset** | **Parent gate** | **HC**  **n=24** | **nPV**  **n=13** | **nnPV**  **n=14** | **p-value** | **BH**  **p-value** | | **p-value** | **BH**  **p-value** |
| Total T cells | Lymphocytes | 72.05 (51.61 - 88.38) | 76.27 (33.33 - 91.36) | 74.60 (55.74 - 87.25) | 0.6875 | 0.9510 | | 0.9051 | 0.9845 |
| Total B cells | Lymphocytes | 6.46 (1.50 - 12.52) | 6.00 (0.55 - 13.76) | 5.96 (0.87 - 31.24) | 0.5680 | 0.8589 | | 0.9051 | 0.9845 |
| Total NK cells | Lymphocytes | 17.90 (2.79 - 41.35) | 11.75 (4.94 - 56.68) | 12.51 (6.83 - 19.15) | 0.1668 | 0.4137 | | 0.4879 | 0.8931 |
| Total ILCs | Lymphocytes | 0.69 (0.28 - 2.13) | 0.72 (0.23 - 2.34) | 0.70 (0.20 - 1.55) | 0.8590 | 0.9510 | | >0.9999 | >0.9999 |
| CD4^+^ T cells | Lymphocytes | 46.40 (22.12 - 65.61) | 39.97 (17.83 - 57.88) | 51.59 (26.86 - 65.59) | 0.8006 | 0.9510 | | 0.3499 | 0.8035 |
| CD8^+^ T cells | Lymphocytes | 15.39 (6.10 - 40.57) | 15.55 (2.24 - 35.67) | 13.91 (5.58 - 28.26) | 0.8297 | 0.9510 | | 0.6848 | 0.9230 |
| Double negative CD4^-^CD8^-^ T cells | Lymphocytes | 1.25 (0.45 - 3.88) | 0.57 (0.20 - 2.76) | 0.82 (0.42 - 1.46) | 0.0020 | **0.0413** | | 0.9051 | 0.9845 |
| Double positive CD4^+^CD8^+^ T cells | Lymphocytes | 0.37 (0.12 - 1.70) | 0.45 (0.06 - 1.68) | 0.56 (0.15 - 1.04) | 0.3938 | 0.6425 | | 0.5186 | 0.8931 |
| γδ T cells | Lymphocytes | 2.09 (0.71 - 9.86) | 2.58 (0.99 - 5.89) | 1.34 (0.74 - 3.73) | 0.2257 | 0.4660 | | 0.0683 | 0.3529 |
| NKT-like cells | Lymphocytes | 5.80 (1.80 - 8.72) | 5.74 (1.20 - 16.91) | 5.61 (2.17 - 16.27) | 0.2046 | 0.4530 | | 0.6848 | 0.9230 |
| Total DCs (CD3^-^CD19^-^CD56^-^) | Lymphocytes | 9.07 (3.82 - 33.28) | 6.55 (1.41 - 9.68) | 5.22 (2.15 - 14.41) | 0.0039 | 0.0602 | | 0.4879 | 0.8931 |
| Classical monocytes | Monocytes | 90.52 (81.90 - 96.90) | 94.86 (87.85 - 99.22) | 92.32 (84.70 - 98.99) | 0.0066 | 0.0602 | | 0.9405 | 0.9999 |
| Intermediate monocytes | Monocytes | 2.94 (0.65 - 11.06) | 1.99 (0.17 - 4.17) | 2.15 (0.47 - 3.97) | 0.0438 | 0.1831 | | 0.6848 | 0.9230 |
| Non-classical monocytes | Monocytes | 5.11 (1.00 - 10.59) | 1.60 (0.05 - 7.69) | 4.08 (0.06 - 9.79) | 0.0068 | 0.0602 | | 0.2388 | 0.7050 |
| Early NK cells | NK cells | 2.12 (0.33 - 7.86) | 1.15 (0.14 - 9.89) | 2.24 (0.78 - 9.06) | 0.2977 | 0.5429 | | 0.1159 | 0.4791 |
| Mature NK cells | NK cells | 70.32 (32.14 - 87.48) | 83.15 (45.60 - 93.71) | 78.94 (36.30 - 85.24) | 0.0232 | 0.1598 | | 0.1408 | 0.4850 |
| Terminal NK | NK cells | 2.60 (1.37 - 6.67) | 3.48 (1.84 - 30.84) | 3.89 (1.20 - 8.55) | 0.0418 | 0.1831 | | 0.8300 | 0.9845 |
| CD4^+^ ILCs | ILCs | 21.91 (5.96 - 44.95) | 12.42 (5.66 - 42.07) | 14.24 (4.57 - 43.49) | 0.0480 | 0.1831 | | 0.9810 | 0.9999 |
| CD4^-^ ILCs | ILCs | 53.81 (23.46 - 74.79) | 57.90 (39.30 - 73.21) | 57.94 (33.98 - 79.71) | 0.0502 | 0.1831 | | 0.7930 | 0.9845 |
| Double negative CD4^-^CD8^-^ T cells | T cells | 1.68 (0.60 - 6.83) | 0.94 (0.41 - 3.32) | 1.08 (0.60 - 1.95) | 0.0010 | **0.0310** | | 0.8300 | 0.9845 |
| Double positive CD4^+^CD8^+^ T cells | T cells | 0.62 (0.18 - 2.28) | 0.63 (0.11 - 2.20) | 0.78 (0.21 - 1.56) | 0.3938 | 0.6425 | | 0.5186 | 0.8931 |
| CD4^+^ T cells | T cells | 63.41 (37.44 - 84.17) | 57.28 (35.54 - 86.49) | 69.56 (44.93 - 84.85) | 0.8006 | 0.9510 | | 0.3499 | 0.8035 |
| Naive CD4^+^ T cells | T cells | 34.16 (7.92 - 50.36) | 28.80 (6.99 - 65.33) | 33.80 (12.00 - 63.67) | 0.7576 | 0.9510 | | 0.7564 | 0.9770 |
| Central memory T cells | T cells | 7.09 (0.01 - 13.82) | 8.71 (5.22 - 12.01) | 8.09 (4.34 - 12.53) | 0.0458 | 0.1831 | | 0.6500 | 0.9230 |
| Effector memory T cells | T cells | 13.60 (0.02 - 36.09) | 8.13 (4.75 - 20.70) | 12.16 (6.07 - 21.49) | 0.1554 | 0.4015 | | 0.1408 | 0.4850 |
| Early like effector memory CD4^+^ T cells | T cells | 4.41 (0.00 - 18.99) | 2.39 (1.74 - 6.04) | 2.86 (0.90 - 6.74) | 0.0115 | 0.0891 | | 0.5186 | 0.8931 |
| Effector effector memory CD4^+^ T cells | T cells | 7.26 (0.01 - 17.00) | 4.11 (1.47 - 13.73) | 7.00 (3.70 - 12.75) | 0.2805 | 0.5270 | | 0.0763 | 0.3639 |
| Terminal effector memory CD4^+^ T cells | T cells | 0.23 (0.00 - 3.03) | 0.55 (0.18 - 5.95) | 0.35 (0.03 - 6.90) | 0.0775 | 0.2403 | | 0.5502 | 0.9220 |
| Regulatory T cells | T cells | 3.94 (2.10 - 6.36) | 4.07 (1.72 - 5.63) | 3.77 (2.61 - 5.25) | 0.3248 | 0.5754 | | 0.9810 | 0.9999 |
| CD39^-^CD45RA^-^ Treg | T cells | 0.82 (0.00 - 2.50) | 0.97 (0.31 - 2.22) | 0.80 (0.32 - 2.51) | 0.7434 | 0.9510 | | 0.2800 | 0.7548 |
| CD39^-^CD45RA^+^ Treg | T cells | 1.12 (0.42 - 2.98) | 1.57 (0.47 - 2.27) | 1.30 (0.68 - 2.05) | 0.8590 | 0.9510 | | 0.5826 | 0.9230 |
| CD39^+^CD45RA^-^ Treg | T cells | 1.73 (0.02 - 2.65) | 1.07 (0.13 - 1.98) | 1.14 (0.45 - 2.55) | 0.0713 | 0.2327 | | 0.1279 | 0.4850 |
| Follicular T helper cells | T cells | 0.73 (0.18 - 2.82) | 0.42 (0.09 - 0.67) | 0.35 (0.09 - 1.45) | 0.0003 | **0.0186** | | 0.4296 | 0.8931 |
| CD8^+^ T cells | T cells | 19.47 (9.43 - 49.31) | 25.17 (4.46 - 39.04) | 17.54 (8.09 - 37.35) | 0.6737 | 0.9510 | | 0.5186 | 0.8931 |
| Naive CD8^+^ T cells | T cells | 2.90 (0.85 - 12.53) | 3.12 (1.21 - 9.41) | 4.41 (1.02 - 24.94) | 0.9329 | 0.9627 | | 0.8300 | 0.9845 |
| Central memory CD8^+^ T cells | T cells | 0.53 (0.00 - 1.71) | 0.65 (0.10 - 1.32) | 0.52 (0.29 - 0.90) | 0.9627 | 0.9627 | | 0.9051 | 0.9845 |
| Effector memory CD8^+^ T cells | T cells | 2.45 (0.01 - 6.66) | 3.67 (0.39 - 6.56) | 2.17 (0.92 - 4.85) | 0.7434 | 0.9510 | | 0.6848 | 0.9230 |
| Early-like effector memory CD8^+^ T cells | T cells | 0.52 (0.00 - 1.18) | 0.48 (0.08 - 1.33) | 0.33 (0.16 - 1.13) | 0.1344 | 0.3623 | | 0.4020 | 0.8901 |
| Effector effector memory CD8^+^ T cells | T cells | 1.23 (0.00 - 3.21) | 1.33 (0.18 - 2.67) | 0.91 (0.43 - 2.69) | 0.7434 | 0.9510 | | 0.6848 | 0.9230 |
| Intermediate effector memory CD8^+^ T cells | T cells | 0.27 (0.00 - 1.00) | 0.26 (0.04 - 1.02) | 0.22 (0.05 - 1.23) | 0.8151 | 0.9510 | | 0.8674 | 0.9845 |
| Terminal effector CD8^+^ T cells | T cells | 0.31 (0.00 - 1.98) | 0.92 (0.09 - 3.82) | 0.19 (0.05 - 1.40) | 0.8151 | 0.9510 | | 0.0850 | 0.3764 |
| CD8^+^TEMRA | T cells | 1.92 (0.18 - 13.66) | 1.59 (0.22 - 7.49) | 1.72 (0.08 - 6.18) | 0.9329 | 0.9627 | | 0.5186 | 0.8931 |
| γδ^+^ T cells | T cells | 2.87 (0.91 - 17.38) | 5.03 (1.24 - 7.33) | 1.87 (1.00 - 4.85) | 0.1979 | 0.4530 | | 0.0107 | 0.0737 |
| CCR7^-^CD45RA^++^ γδ T cells | T cells | 0.64 (0.03 - 8.04) | 0.76 (0.14 - 3.77) | 0.38 (0.04 - 3.15) | 0.0990 | 0.2790 | | 0.1852 | 0.6043 |
| CCR7^-^CD45RA^-^ γδ T cells | T cells | 0.55 (0.00 - 2.96) | 0.52 (0.13 - 2.76) | 0.45 (0.21 - 0.84) | 0.4480 | 0.7119 | | 0.6500 | 0.9230 |
| CCR7^+^ γδ T cells | T cells | 0.28 (0.10 - 0.82) | 0.39 (0.09 - 1.16) | 0.26 (0.16 - 0.67) | 0.8151 | 0.9510 | | 0.2020 | 0.6262 |
| NKT-like cells | T cells | 8.09 (2.31 - 12.11) | 10.23 (2.39 - 20.34) | 8.42 (3.16 - 21.72) | 0.0951 | 0.2790 | | 0.2800 | 0.7548 |
| CD2^+^CD8^+^ NKT-like cells | T cells | 2.73 (0.88 - 8.83) | 4.31 (1.02 - 11.40) | 3.91 (1.53 - 17.88) | 0.2185 | 0.4660 | | 0.7564 | 0.9770 |
| CD2^+^CD8^-^ NKT-like cells | T cells | 1.25 (0.33 - 5.27) | 1.55 (0.36 - 6.31) | 1.88 (0.73 - 4.43) | 0.3533 | 0.6085 | | 0.6500 | 0.9230 |
| CD2^-^ NKT-like cells | T cells | 0.85 (0.19 - 6.56) | 1.13 (0.24 - 8.83) | 0.73 (0.35 - 2.75) | 0.4593 | 0.7119 | | 0.3499 | 0.8035 |
| Naive B cells | B cells | 75.50 (41.37 - 88.69) | 63.93 (7.20 - 81.16) | 83.18 (45.82 - 93.96) | 0.8884 | 0.9627 | | 0.0004 | **0.0041** |
| Double negative (IgD^-^CD27^-^) B cells | B cells | 8.12 (1.73 - 18.84) | 8.38 (3.35 - 33.19) | 4.42 (2.19 - 6.83) | 0.0458 | 0.1831 | | 0.0033 | **0.0292** |
| Transitional B cells | B cells | 9.09 (2.13 - 21.76) | 2.43 (0.81 - 86.59) | 9.66 (0.87 - 39.66) | 0.0346 | 0.1831 | | 0.0107 | 0.0737 |
| Total memory B cells | B cells | 11.28 (3.84 - 51.03) | 27.18 (2.97 - 49.79) | 6.36 (2.26 - 15.29) | 0.9627 | 0.9627 | | <0.0001 | **<0.0012** |
| Unswitched memory B cells | B cells | 3.86 (1.58 - 30.97) | 6.06 (0.19 - 19.95) | 1.16 (0.27 - 3.38) | 0.0438 | 0.1831 | | <0.0001 | **<0.0012** |
| Switched memory B cells | B cells | 4.94 (1.46 - 15.26) | 18.57 (2.67 - 42.19) | 3.38 (1.13 - 11.83) | 0.2330 | 0.4660 | | <0.0001 | **<0.0012** |
| IgG^+^ switched memory B cells | B cells | 2.01 (0.40 - 9.08) | 9.66 (1.68 - 20.91) | 2.12 (0.54 - 6.67) | 0.0549 | 0.1891 | | <0.0001 | **<0.0012** |
| Plasmablasts | B cells | 0.12 (0.00 - 0.68) | 0.14 (0.02 - 0.74) | 0.14 (0.04 - 2.52) | 0.2482 | 0.4809 | | >0.9999 | >0.9999 |
| CD24^hi^CD38^hi^ Breg | B cells | 5.25 (0.48 - 12.92) | 0.30 (0.05 - 81.33) | 3.61 (0.31 - 14.01) | 0.0050 | 0.0602 | | 0.0222 | 0.1251 |
| CD24^hi^CD27^+^ Breg | B cells | 4.71 (1.67 - 39.88) | 15.46 (0.84 - 30.18) | 0.77 (0.21 - 2.98) | 0.1788 | 0.4264 | | <0.0001 | **<0.0012** |
| CD11c^+^ DCs | Dendritic cells | 2.57 (0.65 - 10.74) | 0.85 (0.08 - 11.97) | 5.32 (0.34 - 20.62) | 0.9627 | 0.9627 | | 0.0145 | 0.0899 |
| CD123^+^ pDCs | Dendritic cells | 1.32 (0.19 - 7.39) | 0.52 (0.09 - 4.02) | 1.39 (0.30 - 3.28) | 0.6466 | 0.9510 | | 0.3255 | 0.8035 |

**Supplementary Table 6** – Absolute values of major immune cell populations. Absolute numbers of basophils, monocytes, and lymphocytes were obtained from routine clinical data of patients. Absolute counts for all other populations were calculated by multiplying the absolute lymphocyte count by the frequency of each major immune cell population within the lymphocyte compartment, as defined by the manual gating strategy. T cells were identified as CD3⁺TCRγδ⁺^/^⁻, B cells as CD19^+^CD20^+/-^/CD19^+/-^CD20^+^, natural killer (NK) cells as CD3^-^HLA DR^-^, innate lymphoid cells (ILCs) as CD127^+^HLA DR^-^, NKT-like cells as CD3^+^CD56^+^ and plasmacytoid dendritic cells (pDCs) as CD123^+^ DCs as shown in the manual gating strategy. Comparisons were performed between healthy controls (HCs) and the pemphigus vulgaris (PV) cohort, and between rituximab-naive (nPV) and rituximab-non-naive (nnPV) patients. Mann-Whitney U tests were used for all group comparisons, and p-values were adjusted for multiple testing using the Benjamini-Hochberg false discovery rate correction. No significant differences were observed.

|  |  | **Absolute count 10^6^/L** | | **Mann-Whitney U Test** | |
| --- | --- | --- | --- | --- | --- |
|  |  |  |  | **nPV vs nnPV** | |
| **Subset** | **Source** | **nPV**  **n=13** | **nnPV**  **n=14** | **p-value** | **BH**  **p-value** |
| Basophils | Clinical value | 35.00 (10.00 - 70.00) | 45.00 (20.00 - 90.00) | 0.2117 | 0.6304 |
| Monocytes | Clinical value | 625.00 (390.00 - 1010.00) | 690.00 (410.00 - 940.00) | 0.4696 | 0.7044 |
| Lymphocytes | Clinical value | 1640.00 (740.00 - 3780.00) | 1985.00 (780.00 - 3860.00) | 0.3152 | 0.6304 |
| T cells | Lymphocyte | 2295.00 (1200.00 - 4420.00) | 2700.00 (1650.00 - 4550.00) | 0.4696 | 0.7044 |
| B cells | Lymphocyte | 113.09 (12.66 - 151.86) | 151.81 (10.88 - 295.32) | 0.2740 | 0.6304 |
| NK cells | Lymphocyte | 244.47 (96.85 - 855.86) | 268.26 (57.74 - 480.37) | 0.7045 | 0.8995 |
| Total ILCs | Lymphocyte | 12.82 (4.97 - 30.30) | 12.87 (4.10 - 36.67) | 0.8995 | 0.8995 |
| CD4^+^ T cells | Lymphocyte | 711.14 (269.19 - 1331.31) | 905.74 (209.49 - 1461.09) | 0.2312 | 0.6304 |
| CD8^+^ T cells | Lymphocyte | 230.02 (23.98 - 1142.14) | 196.31 (110.44 - 827.03) | 0.8596 | 0.8995 |
| TCR**γ**δ^+^ T cells | Lymphocyte | 34.50 (22.91 - 115.38) | 27.87 (14.68 - 76.78) | 0.1179 | 0.6304 |
| NKT-like cells | Lymphocyte | 105.94 (12.85 - 639.03) | 93.02 (41.62 - 333.46) | 0.8201 | 0.8995 |
| pDCs | Lymphocyte | 0.72 (0.08 - 5.41) | 1.06 (0.29 - 7.64) | 0.1448 | 0.6304 |

**Supplementary Table 7** – Concentrations of 25 serum analytes measured using the Human XL Cytokine Luminex assay. Comparisons were performed between healthy controls (HCs) and the pemphigus vulgaris (PV) cohort, and between rituximab-naive (nPV) and rituximab-non-naive (nnPV) patients. Mann-Whitney U tests were used for all group comparisons, and p-values were adjusted for multiple testing using the Benjamini-Hochberg (BH) false discovery rate correction. Significant differences (adjusted P < 0.05) are indicated in bold.

|  | **Analyte concentration (pg/mL)**  **median (range)** | | | **Mann-Whitney U Test** | | | | |
| --- | --- | --- | --- | --- | --- | --- | --- | --- |
|  |  |  |  | **HC vs PV** | | | **nPV vs nnPV** | |
| **Subset** | **HC**  **(n = 32)** | **nPV**  **(n = 16)** | **nnPV**  **(n = 16)** | **p-value** | **BH**  **p-value** | **p-value** | | **BH**  **p-value** |
| CD40L | 1382.88 (490.03-8754.46) | 7971.70 (490.03-12367.67) | 8803.56 (6226.75-12245.84) | <0.0001 | **<0.0004** | 0.3226 | | 0.7399 |
| EGF | 305.41 (7.06-752.41) | 379.46 (17.90-565.93) | 370.64 (205.05-432.17) | 0.4115 | 0.6179 | 0.5147 | | 0.7399 |
| CCL11/Eotaxin | 161.38 (23.72-1964.60) | 337.79 (82.79-1189.95) | 349.29 (113.69-1640.37) | <0.0001 | **<0.0004** | 0.7037 | | 0.7399 |
| Flt-3 L | 140.12 (74.27-208.69) | 102.98 (25.58-204.82) | 110.83 (73.23-275.75) | 0.0333 | 0.0699 | 0.4175 | | 0.7577 |
| G-CSF | 33.33 (8.70-53.53) | 46.76 (18.03-78.77) | 52.94 (40.52-64.62) | <0.0001 | **<0.0004** | 0.3458 | | 0.7577 |
| CXCL1/GRO-α | 93.74 (21.29-1035.01) | 126.39 (21.29-1895.98) | 112.15 (53.51-438.45) | 0.2117 | 0.3705 | 0.6760 | | 0.7577 |
| CXCL2/GRO-β | 718.88 (131.51-4113.95) | 775.75 (27.38-4964.35) | 868.39 (106.96-3265.13) | 0.4935 | 0.6335 | 0.3045 | | 0.7577 |
| IL-1RA | 397.76 (266.64-960.12) | 618.36 (317.52-2583.56) | 703.42 (332.56-1335.76) | <0.0001 | **<0.0004** | >0.9999 | | >0.7577 |
| IL-7 | 9.12 (3.08-17.51) | 12.39 (3.08-26.72) | 11.97 (3.08-34.24) | 0.0136 | **0.0317** | 0.7306 | | 0.7577 |
| IL-8 | 10.11 (2.18-140.82) | 8.72 (2.18-129.91) | 11.79 (2.18-41.90) | >0.9999 | >0.9999 | 0.7161 | | 0.7577 |
| IFN-γ | 1.94 (0.49-5.70) | 1.62 (0.49-8.91) | 2.61 (0.99-6.14) | 0.4119 | 0.6179 | 0.1976 | | 0.7970 |
| IP-10 | 105.89 (35.03-219.88) | 86.92 (15.70-430.46) | 142.45 (60.36-289.41) | 0.5144 | 0.6335 | 0.2408 | | 0.8075 |
| CCL2/MCP-1 | 402.40 (136.28-631.75) | 353.96 (62.62-753.35) | 371.35 (117.35-607.71) | 0.4919 | 0.6335 | 0.6689 | | 0.8075 |
| CCL20/MIP-3α | 20.43 (9.93-65.49) | 28.06 (7.19-810.00) | 29.16 (19.85-73.63) | 0.0006 | **0.0018** | 0.2995 | | 0.8075 |
| CCL19/MIP-3β | 68.35 (4.52-469.20) | 70.39 (34.34-470.04) | 111.38 (26.90-142.26) | 0.9814 | 0.9999 | 0.5831 | | 0.8075 |
| PD-L1/B7-H1/CD274 | 130.88 (6.67-266.59) | 178.94 (102.48-814.03) | 174.86 (116.29-297.21) | 0.0016 | **0.0042** | 0.9852 | | 0.8075 |
| PDGF-AA | 10014.10 (3249.11-19494.88) | 9272.54 (1680.73-19494.88) | 11788.65 (3899.26-19494.88) | 0.5430 | 0.6335 | 0.1057 | | 0.8075 |
| PDGF-AB/BB | 3757.10 (969.06-17304.23) | 7057.19 (1329.57-17304.23) | 7819.52 (3374.03-17304.23) | <0.0001 | **<0.0004** | 0.5528 | | 0.8075 |
| CCL5/RANTES | 25756.47 (5783.16-94015.41) | 46179.40 (8310.96-212163.64) | 57011.23 (39539.30-125997.93) | 0.0002 | **0.0007** | 0.1016 | | 0.8075 |
| TNF-β | 5.21 (0.87-9.87) | 3.36 (0.87-8.76) | 6.34 (0.87-9.96) | >0.9999 | >0.9999 | 0.0358 | | 0.9999 |
| VEGF | 254.94 (76.20-1071.80) | 307.36 (92.25-841.04) | 365.03 (148.97-1121.33) | 0.0809 | 0.1544 | 0.3608 | | 0.9999 |

**Supplementary Table 8 –** The frequency of the T cell UMAP-FlowSOM cluster subsets within the total T-cell population in healthy controls (HCs), rituximab-naive (nPV) patients, and rituximab-non-naive (nnPV) patients. Comparisons were performed between healthy controls (HCs) and the pemphigus vulgaris (PV) cohort, and between rituximab-naive (nPV) and rituximab-non-naive (nnPV) patients. Mann-Whitney U tests were used for all group comparisons, and p-values were adjusted for multiple testing using the Benjamini-Hochberg false discovery rate correction. No significant differences were observed. Abbreviations: EM, effector memory; TEMRA, T effector memory CD45RA⁺; TCM, T central memory; Treg, regulatory T cell.

|  | **Frequency (%) in total T cell population.**  **median (range)** | | | **Mann-Whitney U Test** | | | | |
| --- | --- | --- | --- | --- | --- | --- | --- | --- |
|  |  |  |  | **HC vs PV** | | **nPV vs nnPV** | | |
| **Subset** | **HC**  **n=24** | **nPV**  **n=13** | **nnPV**  **n=14** | **p-value** | **BH**  **p-value** | | **p-value** | **BH**  **p-value** |
| CD4^+^ naive | 31.28 (6.51 - 47.94) | 22.68 (4.07 - 58.72) | 25.89 (8.97 - 56.82) | 0.9329 | 0.9329 | | 0.7930 | 0.9524 |
| CD4^+^ CD45RA^dim^CD27^-^CD28^-^CCR7^-^ (EM4) and TEMRA | 0.89 (0.17 - 15.16) | 0.60 (0.12 - 9.87) | 0.37 (0.10 - 13.57) | 0.1190 | 0.2975 | | 0.9524 | 0.9524 |
| CD4^+^ CD45RAdimCD27+CD28+CCR7+/- (TEM1 & TCM) | 6.35 (4.04 - 16.58) | 9.02 (3.75 - 13.26) | 8.68 (3.90 - 11.77) | 0.0295 | 0.2067 | | 0.9430 | 0.9524 |
| CD4^+^ CD45RA^-^CD27^+/-^CD28^+^CCR7^-^ (TEM1/2/3 & TCM) | 24.39 (0.15 - 53.89) | 20.60 (15.52 - 33.70) | 23.57 (13.49 - 36.47) | 0.3938 | 0.7876 | | 0.5502 | 0.9524 |
| CD4^+^ regulatory | 3.11 (0.11 - 6.75) | 2.23 (1.29 - 4.13) | 2.80 (1.43 - 5.44) | 0.0485 | 0.2067 | | 0.2339 | 0.7797 |
| CD8^+^ naive | 4.43 (1.23 - 16.38) | 5.20 (1.49 - 12.36) | 5.20 (2.89 - 28.06) | 0.5713 | 0.9261 | | 0.5826 | 0.9524 |
| CD8^+^ TEMRA | 8.44 (1.05 - 33.74) | 13.60 (1.04 - 33.56) | 6.75 (0.39 - 29.27) | 0.6637 | 0.9261 | | 0.5186 | 0.9524 |
| CD8^+^ effector memory | 9.17 (0.48 - 24.07) | 12.38 (3.31 - 26.75) | 8.25 (3.63 - 16.23) | 0.8335 | 0.9261 | | 0.1546 | 0.7797 |
| Double positive (CD4^+^CD8^+^) | 0.43 (0.14 - 2.60) | 0.49 (0.15 - 1.96) | 0.49 (0.21 - 1.09) | 0.7469 | 0.9261 | | 0.8021 | 0.9524 |
| Double negative (CD4^-^CD8^-^) | 4.53 (1.42 - 21.25) | 4.20 (1.46 - 10.17) | 2.69 (1.27 - 5.77) | 0.0620 | 0.2067 | | 0.1852 | 0.7797 |
